# Regulation of Genome Architecture in Huntington’s Disease

**DOI:** 10.1021/acs.biochem.5c00029

**Published:** 2025-04-27

**Authors:** Stephanie Portillo-Ledesma, Minna Hang, Tamar Schlick

**Affiliations:** † Department of Chemistry, New York University, 100 Washington Square East, Silver Building, New York, New York 10003, United States; ‡ Courant Institute of Mathematical Sciences, New York University, 251 Mercer St., New York, New York 10012, United States; § New York University-East China Normal University Center for Computational Chemistry, New York University Shanghai, Shanghai 200122, China; ∥ Simons Center for Computational Physical Chemistry, New York University, 24 Waverly Place, Silver Building, New York, New York 10003, United States

## Abstract

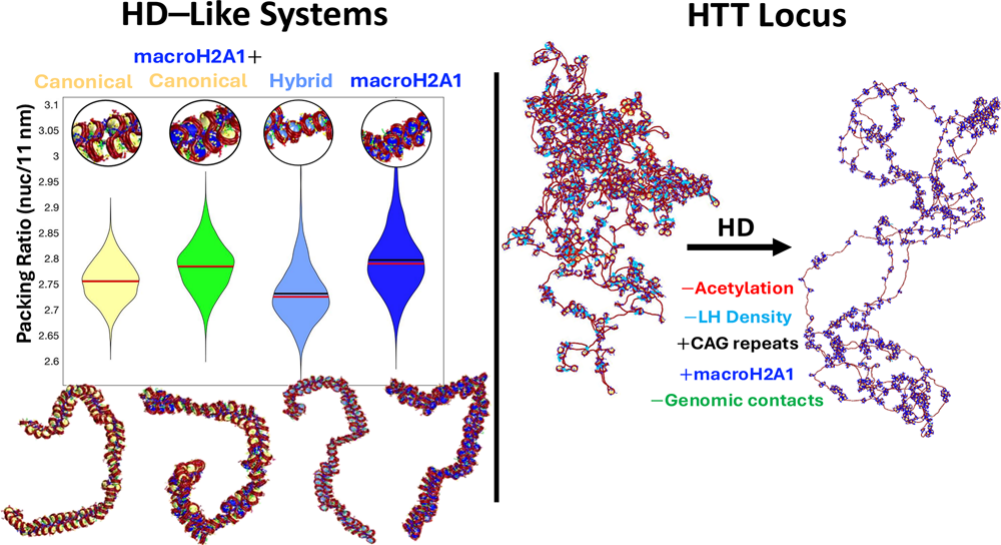

Huntington’s disease (HD) is a neurological condition caused by an excessive expansion of CAG repeats in the Huntingtin (HTT) gene. Although experiments have shown an altered epigenetic landscape and chromatin architecture upon HD development, the structural consequences on the HTT gene remain elusive. Structural data are only available for model nucleosome systems and yeast systems with human nucleosomes. Here, we use our experimentally validated nucleosome-resolution mesoscale chromatin model to investigate folding changes of the HTT gene associated with HD. We investigate how the histone fold domain of the variant macroH2A1, a biomarker of HD, affects the genome structure by modeling HD-like systems that contain (i) 100% canonical, (ii) 100% macroH2A1, (iii) 50% canonical and 50% macroH2A1, and (iv) 100% hybrid cores (one canonical H2A and one macroH2A1 per nucleosome). Then, we model the mouse HTT gene in healthy and HD conditions by incorporating the CAG expansion and macroH2A1 cores, reducing the linker histone density and tail acetylation levels, and incorporating genomic contacts. Overall, our results show that the histone fold domain of macroH2A1 affects chromatin compaction in a fiber-dependent manner (i.e., nucleosome distribution dependent) and can thus both enhance or repress HTT gene expression. Our modeling of the HTT gene shows that HTT is less compact in the diseased condition, which could accelerate the production of the mutated protein. By suggesting the structural biophysical consequences of the HTT gene under HD conditions, our findings may help in the development of diagnostic and therapeutic treatments for HD.

## Introduction

Huntington’s disease (HD) is an inherited progressive neurodegenerative disease caused by an unstable expansion of repetitive short DNA sequences, or short tandem repeats (STRs). More specifically, it is a product of an abnormal expansion of CAG repeats located within the coding sequence of the Huntingtin (HTT) gene.^1^ The extent to which the disease is manifested in patients is related to the number of CAG repeats. Generally, more than 27 CAG repeats is associated with the onset of HD; the wildtype HTT gene usually contains 10–27 repeats.^2^ A range of 26–38 repeats is associated with an HD risk, while beyond 39 repeats is associated with disease.^2^

A high CAG repeat number results in the production of a toxic aggregated mutant htt protein (mhtt) of the HTT gene that affects brain function.^3^

On the functional level, genome studies have shown impaired regulation of genome architecture and its epigenetic control during HD.^4,5^ For example, neuronal and glial development is affected by the HTT mutation due to an impaired epigenetic landscape and chromatin architecture at the level of Topologically Associated Domains (TADs).^6^ Abnormal H3K9 acetylation, important for memory acquisition and recall, is also found in HD.^7^ That histone acetylation levels are reduced in mouse models of HD^8^ also suggests that histone deacetylase (HDAC) inhibitors could be used as therapeutic alternatives.^9^ Indeed, recent studies have shown that HDAC inhibition helps reduce the motor symptoms and transcriptional abnormalities observed in HD.^10,11^

On the structural level of the HTT gene, the CAG expansion level affects local chromatin architecture.^6^ In turn, the altered architecture of the HTT gene might be related to the development of HD.^12^ Indeed, certain chromatin configurations are found in HD but not in healthy individuals, and most importantly, some chromatin configurations are found only in symptomatic HD patients.^12^ Chromosome conformation capture experiments suggest that the excessive CAG repeats become disease-associated when they are located in TAD boundaries,^13^ likely due to the role of TADs in the genome organization.

Overall, these studies point to genome reorganization associated with HD, but details at the kb or nucleosome level are largely absent. High resolution data like MNase-seq or Micro-C that can help decipher the detailed genomic structure are lacking for HD cell types. Instead, model systems containing macroH2A1, a histone variant of the core protein H2A identified as a biomarker of HD^14^ and Parkinson’s disease,^15^ are available for reference.^16^ MacroH2A1 appears to regulate the hippocampus function and memory,^17,18^ and its levels increase with disease progression in HD patients.^14^ This link between a histone variant and HD progression may be a valuable clue for therapeutic development.^14^

Structurally, the macroH2A1 variant is different from the canonical histone H2A^19^; it has an additional C-terminal macro domain connected by a long linker to the histone fold domain.^20^ This C-terminal domain interferes with transcription factor and PolII binding, whereas the histone domain affects chromatin remodeling.^21,22^ This difference points to macroH2A1 as a transcriptional repressor, also supported by the preferential location in the inactive X chromosome.^23,24^ However, macroH2A1 can also activate a subset of genes when located in their transcribed regions,^25^ indicating that gene expression regulation by macroH2A1 is likely context-specific.^26^

Although the additional C-terminal domain has been linked to various functions, the histone fold domain of macroH2A1 alone has shown to be sufficient to direct its localization to the inactive X chromosome^27^ in vivo, as well as cause structural and biochemical distortions to nucleosomes and increase their stability in vitro.^19,28^ The histone fold domain of macroH2A1 also affects nucleosome dynamics, stability, and the orientation of the H2A-H2B dimer in molecular dynamics simulations.^29,30^ Additionally, in humanized yeast systems (yeast genome with human histones), the histone fold domain of macroH2A1 tends to increase the nucleosome repeat length (NRL) by ∼10 to 14 bp on average, and reduce the nucleosome occupancy across gene bodies by ∼13 to 17%, similar to what is found when the C-terminal domain is present.^16^ All these clues make the histone fold domain of macroH2A1 an interesting subject of study.

Here, we apply our validated nucleosome-resolution chromatin mesoscale model^31,32^ to investigate the genome architecture of the HTT gene in relation to HD development by “first-order” model HD systems designed to relate to existing structural data. After studying the role of the histone fold domain of macroH2A1 on chromatin fibers found in humanized yeast systems containing the histone variant (“HD–like fibers”), we model the HTT gene in healthy and HD conditions. We show clear structural consequences of the histone fold domain of macroH2A1 variant that are fiber-topology dependent and reveal that the epigenetic landscape typical of HD favors a less compact HTT gene fold that could be more easily transcribed, further contributing to the HD onset. Such structural insights can help guide future diagnostic and therapeutic tools for HD.

## Materials and Methods

### Chromatin Model

**Figure 1:**
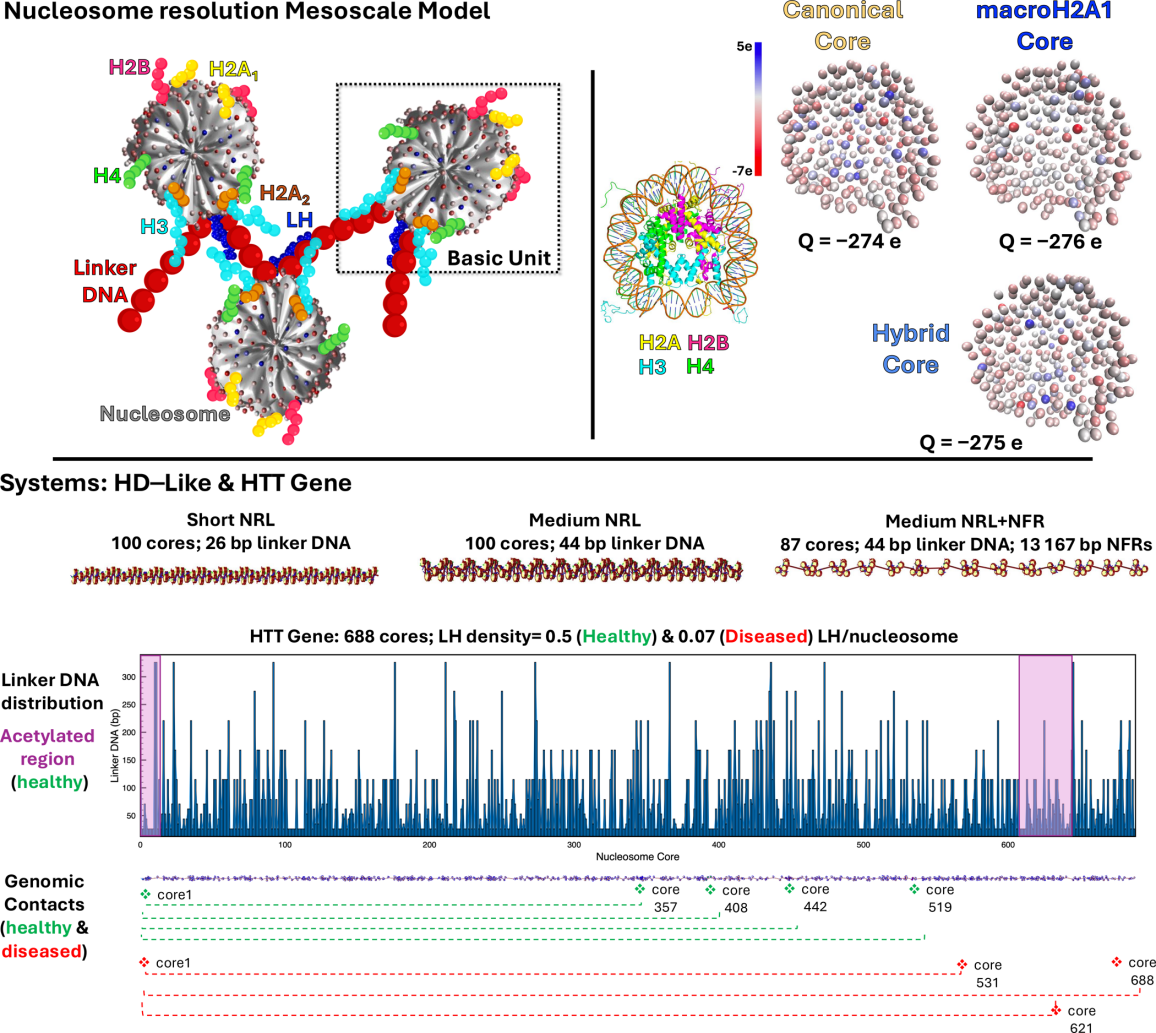
Chromatin mesoscale model and systems. Top left: Nucleosome resolution mesoscale model showing three nucleosomes connected by irregular linker DNA (red beads), details of histone tails (N-terminal tails of H2A, H2B, H3, and H4, and C-terminal tail of H2A (H2A_2_)) and linker histone (blue beads). Top right: 300 DiSCO charges for the canonical, macroH2A1, and hybrid cores. We also show the crystal structure of the nucleosome, indicating the position of each histone, where H2A is shown in yellow. Bottom: Systems studied, including the HD-like systems (Short NRL, Medium NRL, and Medium NRL + NFR, top) and the HTT gene (bottom). For the HTT gene, we show the distribution of linker DNA (Table S3) and the genomic contacts introduced by harmonic restraints between nucleosomes 1 and 357, 408, 442, and 519 in healthy conditions; and between nucleosome 1 and 531, 621, and 688 in the diseased conditions; as well as the acetylation regions covering nucleosomes 1–7 and 629–659, and LH density.

To simulate the structure of the different chromatin systems, we use our nucleosome-resolution mesoscale model (Figure 1) (reviewed in refs (33) and (34) including full functional and parameter details and experimental validations).

Essentially, our model treats nucleosome cores without histone tails as rigid disks with 300 charges distributed in their irregular surfaces enclosing the atomic 3D structure. The charges are calculated from the nucleosome crystal structure using the DiSCO algorithm.^35,36^ In the DiSCO procedure, we model the canonical core based on the nucleosome crystal structure solved at 1.9 Å without the histone tails (PDB 1KX5).^37^ For the macroH2A1 core, we use the crystal structure of the core particle containing two copies of the macroH2A1 histone domain and solved at 3 Å (PDB 1U35).^19^ For the hybrid core containing one copy of each H2A and macroH2A1, we use the crystal structure of the macroH2A1 hybrid core solved at 2.9 Å (PDB 2F8N).^28^ In the crystal structures of the nucleosomes with macroH2A1, some of the other histone proteins are orthologs (*Mus musculus* instead of *Xenopus laevis*) of the histones used to construct the canonical core, but substitution of histone orthologs does not alter the structure and stability of nucleosome cores^38^ as histones are one of the most evolutionary conserved proteins in eukaryotes.^39^ The splicing isoform of macroH2A1 in both crystal structures is the macroH2A1.1. While studying the alternative splicing form macroH2A1.2 would be interesting, the crystal structures are not available.

To each crystal structure without histone tails (the coarse grained tails are later added to the coarse grained core, see below), we add hydrogen and missing atoms using the *tleap* module of AMBER.^40^ We then solvate them with a TIP3P water box, and add Na^+^ and Cl^–^ ions to reach a NaCl concentration of 150 mM. The structures treated with the ff19SB^41^ and bsc1^42^ force fields are then subject to a two stage energy minimization protocol with AMBER^40^ to eliminate unfavorable contacts. First, the water and ions are relaxed, and then the whole system is relaxed. The final relaxed structure without ions and water molecules is used in DiSCO where atomic charges are assigned based on the Amber 1995 force field^43^ to calculate the atomistic electric field with the nonlinear Poisson-Boltzmann solution. Then, 300 Debye–Hückel charges are defined through an optimization procedure that minimizes the error between the electric field of the atomistic nucleosome at distances >5 Å and the Debye–Hückel approximation, as described in detail previously in^36^ and.^35^ Thus, as shown in Figure 1 and Table S1, the macroH2A1 and hybrid cores differ from the canonical core by the positions and values of the 300 discrete charges.

Linker DNAs connecting nucleosomes are treated with a combined wormlike chain and bead model.^44^ The DNA model resolution is approximately ∼9 bp based on the equilibrium distance (*l*_0_) between beads of 3 nm, and the rise between B-DNA base pairs of 0.34 nm. Each bead has a salt-dependent charge calculated based on the charged cylinder approach as developed by Stigter.^45^ By changing the number of linker DNA beads *n*_B_ between nucleosomes, we can simulate linker DNAs of different lengths. The length of the linker DNA connecting nucleosomes, *l*_DNA_, depends on the number of segments that connect linker DNA beads, *n*_S_; the rise between base pairs, *a*; and the equilibrium length, *l*_0_, as follows: *l*_DNA_ = *n*_S_ × *l*_0_/*a*. Thus, for example, for 2–8 *n*_B_ beads, we obtain linker DNAs of 26.47, 35.29, 44.12, 52.94, 61.76, 70.59, and 79.41 bp. We require a minimum of 2 beads for the linker DNA due to the wormlike chain model validity^44^; a fiber with 100 linkers of 1 bead would be governed by topological constraints. We implement the correct nonintegral twist for each DNA segment by first estimating the actual number of turns, τ_nS_, that each DNA linker should make based on its length; we divide the linker length over 10.3 bp/turn, the number of base pairs per turn for DNA in chromatin. For those linker DNA lengths with nonintegral τ_nS_, we add a twist deviation penalty term per DNA segment (φ_nS_) to the total torsion energy based on the difference between the actual number of turns for the DNA linker and the closest integral number of turns. The DNA chain is additionally defined by stretching and bending energy terms. More details on the DNA wormlike chain model parameters and energy functions can be found in^44^ and.^46^

To the surface of the cores, we attach the N-terminal histone tails of each copy of H2A, H2B, H3, and H4, and the C-terminal tail of H2A, all coarse-grained as 5 residues per bead.^31^ Each bead has a total charge equal to the sum of the charges of the residues that form it. For macroH2A1 cores, based on the residue composition, we update the charges of both N-terminal H2A tails by modifying the bead charges from +3, +1, +3, +2 to +1, +3, +2, +1 and both C-terminal H2A tails by modifying the bead charges from +1, 0, +2 to 0, 0, +3 based on the residue sequence.^32^ Similarly, for the hybrid core, we update the charges of the tails for one of the two H2A copies. To simulate fibers containing both canonical and macroH2A1 cores, we add electrostatic and excluded volume energy terms for canonical–macroH2A1 core interactions.

To introduce tail acetylation, we increase the stretching and bending force constants of the histone tails parameters and update the beads coordinates to mimic more folded and rigid tails based on our previous study.^47^ There we saw in all-atom simulations that acetylation produces rigid and folded tails. In our simulation, specific cores and histone tails can become acetylated by a “swap-fold” Monte Carlo move where tails are randomly selected and their coordinates are swapped with those of the corresponding folded version. Folded or acetylated tails do not interact with other chromatin elements due to their compact state.^48^

Linker histone (LH) H1E is also coarse grained as 5 residues per bead. We use 6 rigid beads to describe the globular domain and 22 flexible beads for the C-terminal domain.^49^ Each bead has a charge calculated with the DiSCO algorithm.^35^ As we previously demonstrated, the on- versus off-dyad binding mode of the LH has subtle implications on fiber compaction.^50^ Due to the lack of experimental data here on relevant LH binding in HD–like systems, we limit ourselves to model LHs bound on-dyad.

All beads in the chromatin model (cores, linker DNA, tails, and LHs) interact through electrostatic terms described with the Debye–Hückel potential, and through excluded volume terms described with the Lennard-Jones potential. The total energy contains stretching, bending, twisting, electrostatic, and excluded volume terms with parameters taken from relevant experimental values (e.g., DNA bending and torsional rigidities); details on parameters can be found in Table 5.1 of ref (33), together with all the energy terms.

### Systems

Based on results of humanized yeast systems showing that the histone fold domain of macroH2A1 produces an increase in the nucleosome repeat length (NRL) of ∼15 bp, and a decrease in nucleosome occupancy of ∼13%,^16^ we simulate three “HD–like” systems and the HTT gene (Figure 1):

- Short NRL: 100 cores and NRL = 173 bp
- Medium NRL: 100 cores and NRL = 191 bp
- Medium NRL + nucleosome free regions (NFR): 87 cores, NRL = 191 bp, and 13 NFRs of 167 bp
- HTT gene

The above three “HD–like” systems are simulated in four conditions each: 100% canonical cores, 100% macroH2A1 cores, 100% hybrid cores (that is, one monomer each of macroH2A1 and H2A per core), and a combination of 50% canonical and 50% macroH2A1 cores (Table 1). For the 50–50 canonical/macroH2A1 cores, we distribute macroH2A1 and canonical cores in each HD–like fiber randomly, but we use the same distribution for the Short and Medium NRL fibers of 100 nucleosomes, and a similar distribution for the Medium NRL + NFR fiber of 87 nucleosomes. The distributions can be found in the Supporting Information Table S2.

**Table 1:** Core Composition (in %) in All Systems Studied

All core types				
system	1. canonical (H2A/H2A)	2. macroH2A1 (macroH2A1/macroH2A1)	3. hybrid (H2A/macroH2A1)	4. combination
short NRL	100	100	100	50 canonical
				50 macroH2A1
medium NRL	100	100	100	50 canonical
				50 macroH2A1
medium NRL + NFR	100	100	100	50 canonical
				50 macroH2A1
HTT	100	100	–	–

The HTT gene system is simulated in two conditions: 100% of canonical cores (healthy condition) and 100% of macroH2A1 cores (diseased condition) (Table 1).

The first three “HD–like” systems (Figure 1) are designed to mimic chromatin at increasing HD conditions (CAG repeats) from an initial healthy model to a diseased state. This way, we can investigate the effect that progressive HD conditions have on the fiber structure by first introducing the change in NRL and then adding the effect of decreased nucleosome occupancy. The NRL = 173 bp is selected as it is the closest NRL we can model to describe the average NRL found in yeast cells (165 bp).^51^

The HTT gene (Figure 1) that we model occupies the region of chr5:34,761,744–34,912,534 in the mm10 mouse genome. Nucleosome positions were obtained from MNase-seq data of mouse neural progenitor cells.^52^ In particular, nucleosome positions as obtained with the DANPOS algorithm^53^ were extracted from the database NucMap.^54^ Only those nucleosomes whose positions were at ≥ – 20 bp of their neighbor nucleosomes were retained. The linker DNA lengths obtained from the experimental nucleosome positions are converted to the closest integer value of linker DNA beads with our ∼ 9 bp per segment resolution, with the shortest linker DNA set as 2 beads.^46^ In Table S3 of the Supporting Information, we list the values for the linker DNA beads used in our healthy and HD HTT gene models, including bp units. Positions of histone tail acetylation were obtained from Chip-seq data of mouse hippocampus^8^ that show two acetylation islands in the healthy mouse but no acetylation in the HD mouse. The linker histone density was set as 0.5 LH/nucleosome in the healthy state, with LHs randomly distributed in each of the 30 copies. This LH density has been determined in neuronal cells.^51^ In the diseased state, we reduce LH density 7-fold to 0.07 LH/nucleosome as this has been observed in Spinocerebellar ataxia type 7, a neurodegenerative disease also produced by the unstable expansion of STRs.^55^ In the diseased state, to mimic the expansion of CAG repeats, the HTT gene is expanded in exon 1 by increasing the length of the first linker DNA by 106 bp (∼35 extra repeats). Finally, we introduce genomic contacts as determined with chromosome conformation caption (4C) experiments in healthy and HD patients.^12^ These experiments show that the HTT anchor region located at the beginning of the gene establishes 4 looping interactions in healthy individuals but 3 in symptomatic HD patients. To mimic such chromosome interactions, we incorporate for the HTT gene harmonic restraints between nucleosome cores, similar to our GATA-4 gene modeling.^56^ For the gene in healthy conditions, we add 4 harmonic restraints between core 1 and cores 357, 519, 408, and 442, and for HD conditions, we add three harmonic restraints between core 1 and cores 531, 621, and 688 (Figure 1). The length of these loops mimic the length of the loops found in healthy and HD patients.^12^ The harmonic energy term for each restraint has the form *E*_R_ = *k*(*l* – *l*_0_)^2^, where *k* is a force constant between the two nucleosomes, *l* is their instantaneous distance, and *l*_0_ is the equilibrium distance. We set *k* to 40 kcal/mol/nm and *l*_0_ to 50 nm.

### Sampling

Chromatin fibers are sampled with equilibrium Monte Carlo simulations at temperature 293 K and NaCl concentration of 150 mM. A global pivot move is used to move linker DNA beads or cores by randomly choosing them, selecting a random axis passing through them, and then rotating the shorter part of the fiber about this axis.^32^ Similarly, local translation and rotation moves of the cores and DNA beads are used by randomly selecting an element, choosing a random axis passing through it, and then shifting or rotating the element along the axis.^32^ The three moves are accepted or rejected based on the Metropolis criterion.^57^ Tails are sampled with a regrowth move^32^ using the Rosenbluth scheme.^58^ Linker histone beads of the C-terminal domain are sampled with local translational moves.^49^

We simulate the 100 and 87 nucleosome systems (Short NRL, Medium NRL, and Medium NRL+NFR) for 40 million Monte Carlo steps and with 60 replicas each to ensure convergence (see convergence plots in Figure S1). The replicas are started wtih a different random seed and a residual DNA twist value of 0, −12°, or +12° that mimics natural variations.^59^

Because our model of the HTT gene is quite large, 688 nucleosomes, we simulate the HTT gene in healthy and diseased conditions for 70 million Monte Carlo steps with 30 replicas, also started with a different random seed value and different DNA residual twist value.

### Analysis

- Packing Ratio: The fiber packing ratio is calculated as
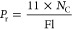
1where *N*_C_ is the total number of nucleosomes and Fl is the fiber length calculated by defining the fiber axis (**r**^*ax*^) with a cubic smoothing spline interpolation to the nucleosomes *x*, *y*, and *z* coordinates; see details in the Supporting Information of ref (60).
- Sedimentation coefficient: Sedimentation coefficients are calculated as
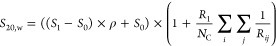
2where *S*_0_ and *S*_1_ are the sedimentation coefficients of a mononucleosome without LH (*S*_0_ = 11.1 *S*)^61^ and with LH (*S*_1_ = 12 *S*),^62^ respectively, ρ is the LH density on the fiber, *R*_1_ is the radius of a nucleosome (*R*_1_ = 5.5 nm), *N*_C_ is the number of nucleosomes in the chromatin fiber, and *R*_*ij*_ is the distance between the nucleosomes *i* and *j*.
- Radius of gyration: The radius of gyration, which describes the overall dimension of the chromatin fiber, is measured as the root mean squared distance of each nucleosome from the center of mass according to
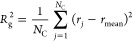
3where *N*_C_ is the number of nucleosomes, *r*_*j*_ is the center position of the nucleosome core *j*, and *r*_mean_ is the average of all core positions
- Internucleosome interactions: Interactions among nucleosomes are calculated in nucleosome resolution for the 6000-configurational ensembles of the Short NRL, Medium NRL, and Medium NRL + NFR systems. Two nucleosomes *i* and *j* are considered to be in contact if any element of nucleosome *i*, such as core, tails, or linker DNA, is less than 2 nm from any element of nucleosome *j*. Internucleosome interaction matrices at nucleosome resolution are decomposed into one-dimensional plots that depict the magnitude of *i*, *i* ± *k* interactions, or contact patterns, according to
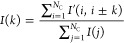
4where *N*_C_ is the number of nucleosomes, *I* is the internucleosome interaction matrix, and *k* is the number of nucleosomes between cores *i* and *j*. For the HTT gene system, internucleosome interactions are calculated every 100,000 Monte Carlo steps during the simulation of each of the 30 copies. Contacts are normalized across each trajectory and summed to create an ensemble contact matrix in bp resolution. This matrix is used to plot contact frequency across the genomic distance and to determine interactions between different epigenetic regions.
- Geometric parameters: We measure the euclidean distance between nucleosome *i* and *i* + 1, the angle between nucleosomes *i*, *i* + 1, and *i* + 2, and the twisting angle between the planes of nucleosomes *i* and *i* + 1.
- Clustering analysis: We use the DBSCAN algorithm to calculate the number of nucleosome clutches and the average number of nucleosomes per clutch, similar to what we did before.^63^ Briefly, we calculate the euclidean distance among all pairs of nucleosomes and then perform the DBSCAN clustering by selecting 3 as the minimum number of nucleosomes needed to form a clutch and 30 nm as the radius of search.
- Persistence length: The fiber persistence length is calculated by fitting an exponential function to the angle defined by two unit tangent vectors of the fiber axis parametric curve (**r**^*ax*^(*i*)) as
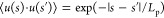
5where *u*(*s*) and *u*(*s′*) are a tangent vector at the beginning of the curve and a tangent vector that correspond to the highest bending, and |*s* – *s′*| is the contour length of the whole fiber.
- End-to-end distance: For the three HD–like systems, we measure the euclidean distance between the first and last nucleosome.
- Tail interactions: We calculate the frequency of interactions of each tail, H2A(macroH2A1) N-terminal, H2A(macroH2A1) C-terminal, H3 N-terminal, H2B N-terminal, and H4 N-terminal with other chromatin elements such as parental cores and DNA, nonparental cores and nonparental DNA, and other tails. Two elements are considered to be in contact if the distance between them is less than 2 nm. Interactions are normalized so the frequency of interaction of each tail with all other elements plus the frequency of being “free” equals 1. We then calculate the ratio between the frequency obtained with canonical cores and the frequency obtained with macroH2A1 cores.

## Results

### Histone Fold Domain of MacroH2A1 Modulates Fiber Architecture and Compaction Differently Depending on the Fiber Topology

**Figure 2:**
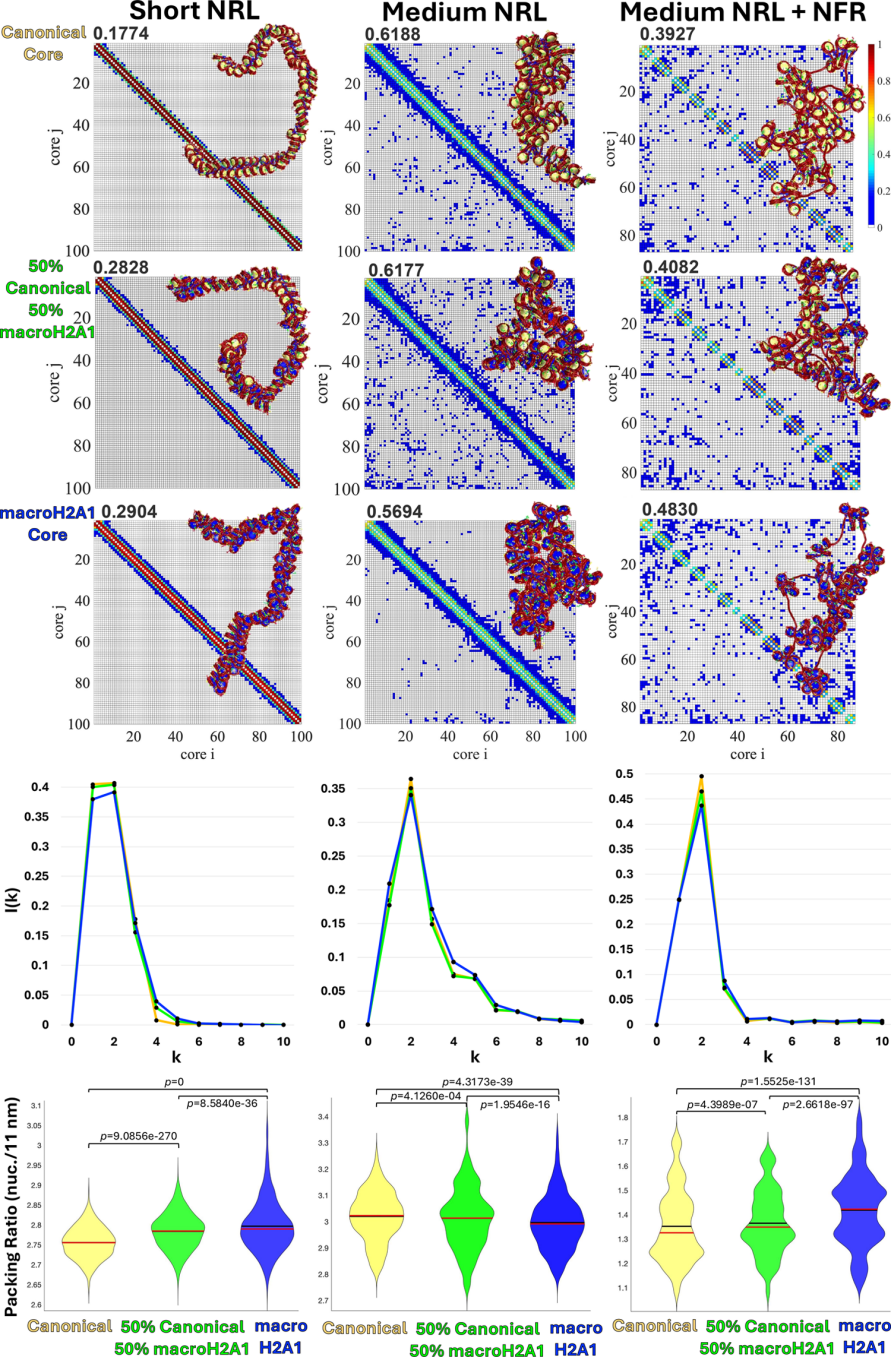
Effect of the macroH2A1 variant on chromatin fiber architecture. For each system, Short NRL, Medium NRL, and Medium NRL + NFR, with all-canonical (yellow), all-macroH2A1 (blue), or a combination of 50% canonical and 50% macroH2A1 (green) cores, we show the internucleosome contact maps calculated from an ensemble of 6000 structures with their corresponding density on the top left corner, representative chromatin configuration, randomly selected, on top of the contact maps with canonical cores yellow and macroH2A1 cores blue, internucleosome interaction plots in 1D, and violin plots for packing ratio, where black lines indicate the mean and red lines indicate the median. Statistical significance: Student’s *t* test.

We begin by studying the folding of the three HD–like systems that represent increasing manifestations of HD: Short NRL, Medium NRL, and Medium NRL + NFR, containing all canonical or all macroH2A1 cores (see Materials and Methods and Table 1). For fibers with canonical cores, the topologies at increasing NRL and/or NFRs (Figure 2) agree with our prior computations^46,60,63,64^ and experimental data.^65−67^ That is, short NRLs create a ladder-like fiber with no long-range interactions, whereas longer NRLs produce a more globular structure with bent linker DNAs and fold into hierarchical loops with an overall zigzag topology. On the other hand, NFRs divide the chromatin fiber, separating genome regions and eliminating short and medium-range (*i* ± 1, 2, 3) contacts near their genomic locations due to stiff and long segments of DNA in the nucleosome depleted region.

A nucleosome core containing a histone variant can interact differently with other cores, or with other chromatin elements like the linker DNA, LH, or tails, producing a different fiber folding and compaction.

For each system, we calculate internucleosome contact maps as a measure of overall fiber organization, a computational analog of Hi-C. As Figure 2 shows, macroH2A1 cores affect the contact patterns compared to the canonical cores. In all three systems, macroH2A1 cores produce higher short-range contacts close to the diagonal, but for the Medium NRL system there is a reduction of medium and long-range contacts. The 1D plots in Figure 2 show that macroH2A1 cores reduce *i* ± 2 interactions in all systems, indicating that the zigzag topology of the fibers is affected. For the Medium NRL system, additionally, an increase of *i* ± 1 interactions is seen, indicating a more disordered and open structure where consecutive nucleosomes are most likely to interact with each other.^65,68,69^ These results indicate that this histone variant modulates the way cores interact with each other and the fiber’s global folding.

**Figure 3:**
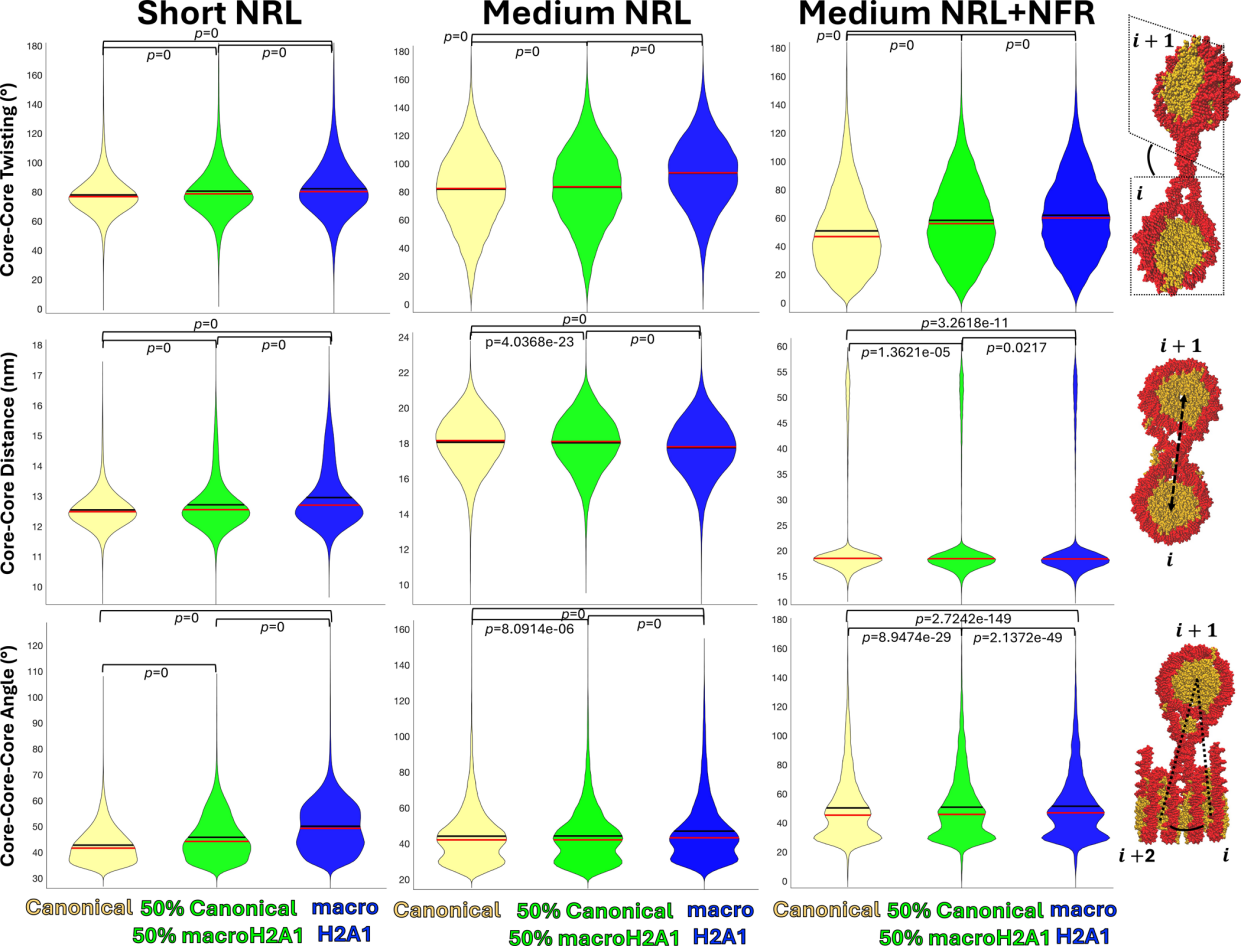
Effect of the macroH2A1 variant on fiber local geometry. For each system, Short NRL, Medium NRL, and Medium NRL + NFR with all-canonical (yellow), all-macroH2A1 cores (blue), or a combination of 50% canonical and 50% macroH2A1 cores (green), we show the angle between the plane of two consecutive cores (core–core twisting), the distance between two consecutive cores (core–core distance), and the angle between three consecutive cores (core–core–core angle) calculated from an ensemble of 6000 structures. For each property, we illustrate at right what is calculated. Black lines in the violin indicate the mean and red lines indicate the median. Statistical significance: Student’s *t* test.

When analyzing the fiber local geometry in Figure 3, we see that parameters that define the fiber architecture also change when cores contain the macroH2A1 variant. For example, the angle between consecutive nucleosome planes increases in all three systems, indicating that the histone fold domain of macroH2A1 affects the orientation of the nucleosome cores in the fiber. This change indicates that macroH2A1 reduces the zigzag topology of the fibers, in agreement with the reduction of *i* ± 2 contacts seen in Figure 2. The largest changes are captured for the Medium NRL system, which shows a decrease in the packing ratio and an increase of *i* ± 1 contacts (Figure 2). On the other hand, the distance between consecutive cores increases for the Short NRL and Medium NRL + NFR systems, but decreases for the Medium NRL system. The distance decrease in the Medium NRL system agrees with the increase of *i* ± 1 contacts and decrease of the packing ratio, indicating a more disordered and open fiber where consecutive nucleosomes in the chromatin chain are closer to one another due to a more unstructured chromatin architecture.^65,68,69^ Finally, the angle formed between three consecutive cores increases for all three systems, with the smallest changes found for the NRL system. This indicates a decrease in the zigzag topology, and agrees with the decrease of *i* ± 2 contacts found for the three systems in Figure 2. Thus, depending on the system configuration, the histone fold domain of macroH2A1 produces different effects on the fiber’s local geometry.

Because the macroH2A1 histone variant is related to the activation and repression of genes,^70^ we also investigate compaction parameters in the HD–like systems. Figure 2 shows the packing ratio for the three systems. We see that for both Short NRL and Medium NRL + NFR, macroH2A1 cores increase fiber compaction, producing fibers with higher packing ratio. On the other hand, for the Medium NRL system, macroH2A1 cores reduce the packing ratio, indicating lower level of condensation.

Similar trends are observed for global compaction parameters, like sedimentation coefficient and radius of gyration (Figure S2). Namely, the Short NRL and Medium NRL + NFR systems exhibit higher sedimentation coefficients and lower radii of gyration when all cores are macroH2A1, indicating a more globally folded fiber. For the Medium NRL system, upon macroH2A1 addition we see lower sedimentation coefficient and higher radius of gyration, indicative of a more open fiber. This global opening agrees with the reduction of medium and long-range contacts found in the contact map of the NRL system with all macroH2A1 cores (Figure 2).

The Medium NRL fiber that exhibits lower compaction with macroH2A1 cores, also shows an increase of *i* ± 1 contacts (Figure 2) and a decrease of *i* ± 1 distances (Figure 3), both suggestive of a more disordered and open fiber.^65,68,69^ This system also exhibits the largest decrease of tail nonparental core interactions (Figure S3), which are essential for fiber folding and compaction.^31^

The two systems where macroH2A1 increases compaction, Short NRL and Medium NRL + NFR, differ from the Medium NRL system in terms of flexibility; they are more rigid due to short linker DNAs or long stiff DNA segments created by nucleosome depleted regions. Thus, we also study changes in persistence length. Overall, we see that macroH2A1 significantly reduces the persistence length of the Short NRL and Medium NRL + NFR systems but not so much the persistence length of the Medium NRL system (Figure S4). Thus, in more rigid fibers, macroH2A1 enhances the conformational flexibility of the chromatin fibers, allowing for higher compaction. The more flexible Medium NRL fiber can better accommodate the changes induced by macroH2A1. Such more flexible fibers with longer linkers could be less sensitive to the effect of the histone variant. Indeed, we studied a fiber with long 61 bp linkers and found no differences in packing ratio between canonical and macroH2A1 cores (Figure S4).

Overall, these results indicate that the histone fold domain of the macroH2A1 variant could repress or activate genes depending on the fiber configuration, providing a possible explanation for the puzzling experimental observations that show both trends.^25,26^

### Combining Canonical and MacroH2A1 Cores in a Single Fiber Shows Transformation of Healthy into Disease Type Fibers

Thus far, we modeled the HD–like systems with all canonical or all macroH2A1 cores. Because the levels of macroH2A1 increase progressively with desease progression in HD,^14^ we additionally studied the three HD–like systems with a combination of 50% macroH2A1 cores and 50% canonical cores. As shown in Figure 2, chromatin packing ratio gradually increases for the Short NRL and Medium NRL + NFR systems, and gradually deacreases for the Medium NRL system when we increase the level of macroH2A1 from 0, to 50, and to 100%. Similar trends are observed in the 1D plots of internucleosome interactions (Figure 2). The peak at *i* ± 2 gradually decreases in the three systems, indicating that addition of macroH2A1 reduces the zigzag topology of the fiber. Fiber local geometry in Figure 3 also shows a gradual change of the three parameters (core–core twisting and distance, and core–core–core angle) as we gradually increase the level of macroH2A1 from 0 to 100%. Overall, these results could reflect the transformation of chromatin from a healthy state into a diseased state during HD progression. While these results were obtained for a random distribution of macroH2A1 and canonical cores, a uniform distribution where both cores are positioned on every other nucleosome produces similar compaction results (Figure S5). Thus, different spread distributions of macroH2A1 cores do not seem to change macroH2A1 effect on fiber compaction.

Because it is not known whether only one or both copies of the canonical H2A histone are substituted by macroH2A1 in the nucleosome, and previous results showed that in vitro macroH2A1 preferentially forms hybrid nucleosomes that combine one copy of H2A with one copy of macroH2A1,^28^ we also studied the three HD–like systems with all hybrid cores, that is, an H2A heterodimer of one H2A and one macroH2A1 per core.

**Figure 4:**
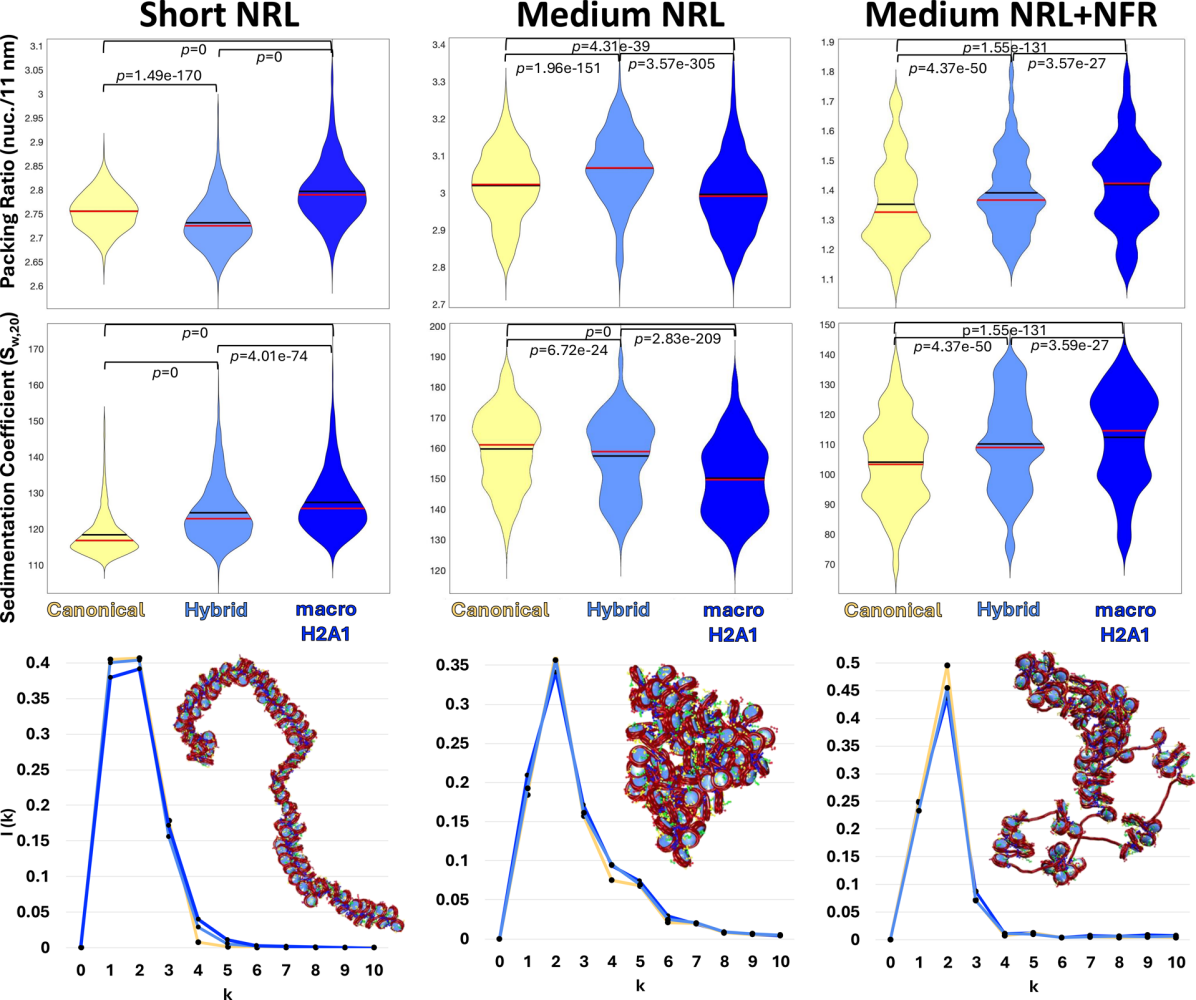
Effects of compaction and internucleosome interactions for systems with hybrid cores (one H2A monomer and one macroH2A1 monomer per core) compared to all-canonical or all-macroH2A1 cores. For each system: Short NRL, Medium NRL, and Medium NRL + NFR with all-canonical cores (yellow), all-hybrid cores (light blue), and all-macroH2A1 cores (blue), we show from top to bottom: violin plots for the packing ratio; violin plots for the sedimentation coefficient; internucleosome interaction plots in 1D and representative fiber configurations of systems with all hybrid cores (light blue). Statistical significance: Student’s *t* test.

As shown in Figure 4, the packing ratio of Short NRL fibers with all hybrid cores is smaller than for all canonical or all macroH2A1 cores. On the other hand, a higher packing ratio is obtained for the medium NRL system containing all hybrid cores than with all canonical or all macroH2A1 cores. Finally, the Medium NRL + NFR system shows an intermediate value of packing ratio with all hybrid cores, with higher compaction than for all canonical cores but lower compaction than for all macroH2A1 cores, similar to the trend observed for the fiber combining 50% canonical with 50% macroH2A1 cores (Figure 2).

These results indicate that hybrid cores behave differently from the homogeneous canonical and macroH2A1 cores, and are not just intermediates. They also agree with molecular dynamics simulations comparing canonical, macroH2A1, and hybrid cores that show that hybrid nucleosomes have dynamic and structural properties that differ from the homogeneous systems.^30^

When analyzing internucleosome interactions, we see for the three HD-like systems that the peak corresponding to *i* ± 2 interactions falls between those of all canonical and all macroH2A1 cores, but they are closer to the values of all canonical cores in the Short and Medium NRL systems, and closer to the value of all macroH2A1 cores in the Medium NRL + NFR system.

Interestingly, global compaction parameters like the sedimentation coefficient shown in Figure 4 indicate that HD–like fibers with all hybrid cores have a global folding compaction that falls between the fiber with all-canonical and all-macroH2A1 cores. To further inspect these global changes, we study the fiber persistence length and end-to-end distance (Figure S6). For the Short NRL and Medium NRL + NFR systems, macroH2A1 reduces the persistence length and the end-to-end distance significantly. The effect is additive for the number of macroH2A1 copies. Namely, the persistence length and end-to-end distance values gradually decrease as we transition from canonical to hybrid (one copy macroH2A1), and further to macroH2A1 (two copies of macroH2A1) cores. For the Medium NRL system, which shows a decrease in the sedimentation coefficient, macroH2A1 cores slightly decrease the persistence length of the fiber, though the changes are minimal. In terms of end-to-end distance, a clear trend emerges: there is a gradual increase of this distance from canonical, to hybrid, to macroH2A1 cores. Thus, macroH2A1 produces more bent fibers for short linkers or long stiff segments of nucleosome-depleted DNA than canonical systems, but not for 44 bp linkers.

These trends are consistent with effects of linker DNA length where variations can increase the fiber sedimentation coeffient but reduce the packing ratio.^46^ The packing ratio indicates the accessibility to the genetic material, while the sedimentation coefficient reflects the global shape of the fiber. Thus, hybrid cores might behave as intermediates of canonical and macroH2A1 cores at a global level but behave differently on the local level when the asymmetry of the core plays a role in internucleosome interactions.

In relation to HD, these results suggest that the effect of the histone fold domain of macroH2A1 on gene architecture will be different when only one unit of H2A per nucleosome is substituted by macroH2A1 compared to two macroH2A1 copies. Thus, the substitution of one versus two copies of macroH2A1 introduces heterogeneity and provides an epigenetic regulation factor during disease development and progression.

### HD Conditions Favor Transcription of the HTT Gene

Genomic studies of HD point to an epigenetic abnormality of neuronal and glial specific genes, as well as memory related genes.^6,7^ This indicates that epigenetic conditions associated with HD might alter the expression of the HTT gene, further contributing to the disease progression.

To understand in greater detail how the HTT gene is regulated during HD, we model the HTT gene in healthy and diseased conditions (Figure 1) using experimental information on nucleosome positions, epigenetic marks like histone acetylation and LH, and genomic contacts. We also use macroH2A1 cores for the diseased condition and canonical cores for the healthy condition.

**Figure 5:**
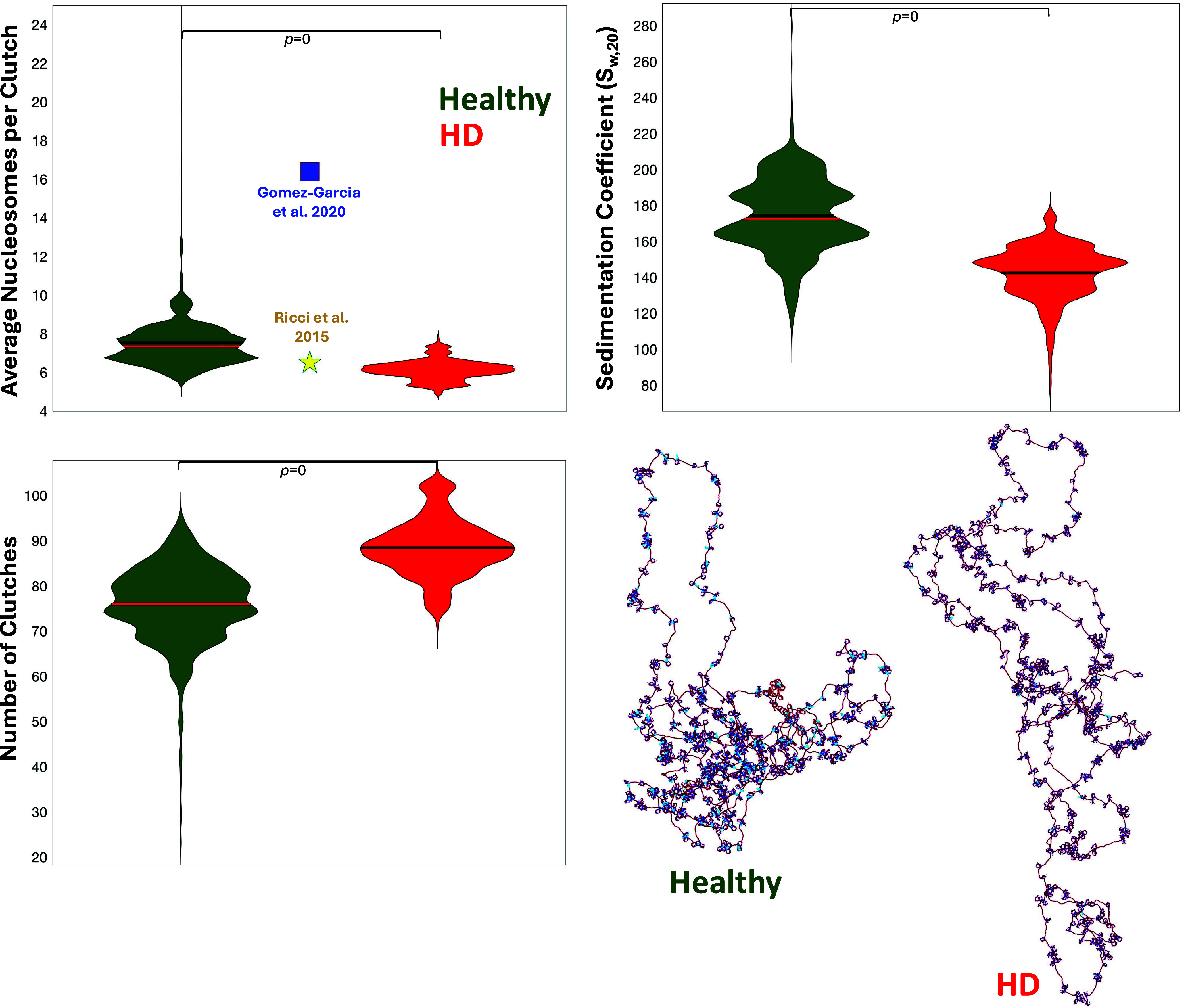
Epigenetic landscape typical of HD favoring transcription of HTT. For the HTT gene system in healthy and diseased conditions, we show violin plots for the sedimentation coefficient and for the average number of nucleosomes per clutch and number of clutches computed from our models. For the average number of nucleosomes per clutch, we also report previous experimentally determined value at the genome-wide level (yellow star^71^) and theoretically determined value at a single-locus level (blue square^72^). Representative structures of the gene in each condition as computed by our models are illustrated, with LHs in cyan, wildtype tails in blue, and acetylated tails in read. Statistical significance: Student’s *t* test.

Figure 5 shows the sedimentation coefficient, nucleosome clutch analysis, and representative fiber configurations for the HTT gene in healthy and diseased conditions. We see that the gene is more open, with a lower sedimentation coefficient and smaller nucleosome clutches, for the diseased state. This effect is in contrast to the more closed and compact HTT gene folds in healthy conditions (Figures 5 and S7). Importantly, the average numbers of nucleosomes per clutch found for both conditions, 7.5 ± 1.5 and 6.1 ± 0.5, are remarkably similar to what has been found previously with super resolution microscopy at a genome-wide level (∼6.5)^71^ and close to mesoscale modeling results obtained at a single locus level (16.4 ± 11.3)^72^ for mouse neural progenitor cells (Figure 5); the latter is the same cell type we use here to set up our HTT gene systems.

**Figure 6:**
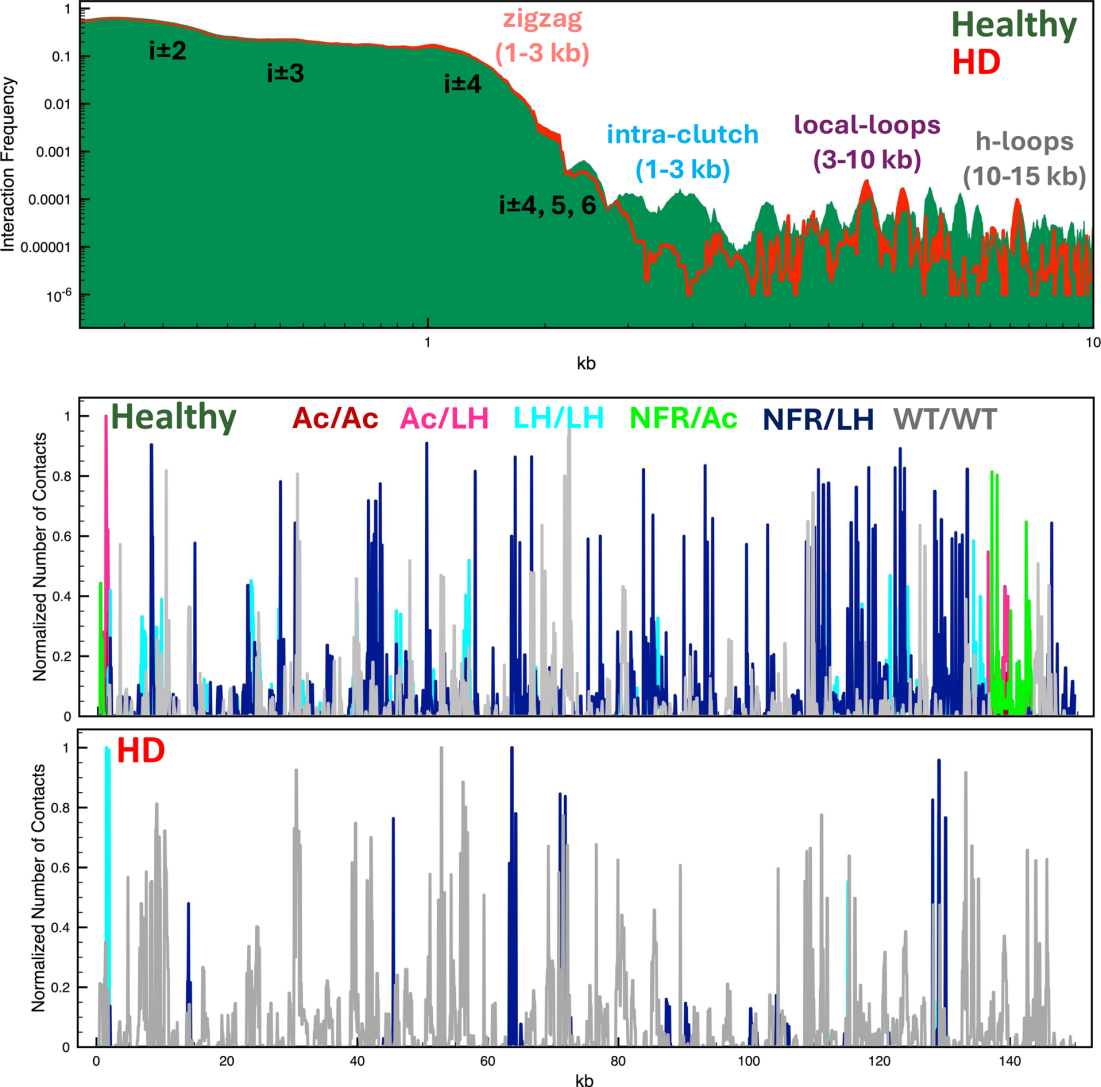
Epigenetic effects of HD on the type of internucleosme interactions in the HTT gene. For the HTT gene system in healthy and diseased conditions, we show at the top internucleosome interaction contacts as a function of the genomic position. We annotate peaks for short-range interactions (*i* ± 2, 3, 4, 5, 6) and structural motifs like zigzag topology, clutches, and hierarchical loops. At bottom, we show in two plots the number of contacts established in the HTT gene between acetylated regions (Ac/Ac, red), an acetylated region and LHs (Ac/LH, pink), two LH regions (LH/LH, cyan), nucleosome free regions and acetylated regions (NFR/Ac, green), nucleosome free regions and LHs (NFR/LH, blue), and between two regions that have no epigenetic marks (WT/WT, gray).

Internucleosome interactions in Figure 6 show that healthy conditions favor interactions at medium range typical of nucleosome clutches (1–2 kb range), loops between neighboring clutches (5–10 kb), and hierarchical loops (10–20 kb). This is reflected by more folded and compact fibers (see Figures 5 and S7). On the other hand, higher short-range interactions (∼1 kb) and much more open architecture occur in the diseased condition.

To determine how epigenetic marks drive gene folding in each condition, in Figure 6 we dissect the internucleosome contacts into interaction types. We see that in healthy conditions more interactions result from the higher LH density, such as LH/LH, NFR/LH, and to a lesser extent Ac/LH. On the other hand, the HTT gene in the diseased condition exhibits mostly interactions between regions without any epigenetic mark (WT/WT type interactions).

We previously showed for the HOXC gene that interactions between LH/Ac regions help gene folding and create a contact hub.^33^ Thus, the reduction of LH density and lack of acetylation islands in the HTT gene in diseased conditions combine to decrease the internucleosome contacts, affecting global fiber folding and compaction. Although the HTT gene contains one more restraint at healthy conditions compared to the diseased conditions, we expect the difference in compaction to remain due to the higher LH density and the two acetylation islands in the healthy fibers. A higher LH density helps compact the chromatin fiber by increasing favorable electrostatic interactions^50^; acetylation islands bring together acetylated regions^73^; and LH-rich and acetylated regions create long-range contacts.^48,74^

An overall more open structure for the HD condition (Figure 5) agrees well with 3D models of a 2 Mb chromatin region containing the HTT gene in wildtype and HD contexts we show in Figure S8.^6^ Alcalá-Vida et al. used 4C-seq experimental genomic contacts of female healthy and HD mouse models to generate the folded genome structures. When comparing the total number of 4C contacts, both female and male mice show more contacts for healthy versus HD systems, in agreement with our results. However, only the female mice sample shows a higher score for the total number of contacts in the healthy state. This could be related to gender-related differences associated with HD: females tend to have a less favorable outcome of the disease than males.^75^

These results indicate that in HD, the epigenetic landscape – namely histone acetylation levels, LH density, and the histone fold domain of the macroH2A1 variant, as well as genomic interactions and the CAG expansion– modulates the HTT architecture to produce a more open and accessible gene. In such conditions, a higher expression of the mutated htt protein is favored, which in turn accelerates disease progression. This agrees with murine HD models showing that mhtt protein levels in the brain and cerebrospinal fluid increase later in life,^76^ and with human HD samples, showing an increase of the mhtt protein in the cerebrospinal fluid with disease stage.^77^ However, in HD mouse models of 6 months, the mRNA levels of htt are slightly reduced compared to the healthy mouse.^6^

## Discussion

Huntington’s disease (HD) is a progressive neurodegenerative disorder that affects approximately 41 thousand people in the United States and more than 200 thousand are at risk of inheriting it. Because HD arises from a mutation on the HTT gene, understanding the associated changes in genome architecture can help develop new therapeutic avenues to diagnose and alleviate it.^78,79^ Thus far, there have been few therapies that successfully treat this disorder. Recent therapeutic strategies have focused on lowering the levels of the mhtt protein,^80^ but more fundamental approaches are needed. Thus, understanding the regulation of the mutated HTT gene during HD could help develop genetic tools to inhibit its initial transcription.

Here, we have used our nucleosome-resolution mesoscale chromatin model to investigate the HTT structural genomic rearrangements associated with HD development and progression with model systems. In particular, we focused on the role of the histone fold domain of the macroH2A1 variant, a biomarker of HD.^14^

Based on experiments showing that the histone fold domain of macroH2A1 increases linker DNA length and reduces nucleosome occupancy,^16^ we designed chromatin fibers typical of yeast cells that have short and medium DNA lengths and included nucleosome free regions (NFRs). The results show that depending on the fiber configuration (linker DNA length and NFRs), the histone fold domain of macroH2A1 can either increase or decrease chromatin compaction. This dual potential agrees with gene expression studies showing that macroH2A1 can activate or repress genes,^25,26^ and that in HD, some genes of neuronal and glial cells increase their expression while others decrease it.^6^ The precise values of NRL and NFRs in the genes activated by macroH2A versus repressed is unknown. Experiments indicate that single well-positioned macroH2A nucleosomes are enriched on all gene promoters, and that extended, large chromatin domains (few kbp) containing mostly fuzzy macroH2A nucleosomes are located up or downstream of low or non expressed genes.^70^ Other experiments on cancer cells indicate that macroH2A is enriched at the TSS of activated genes and/or at the promoter and gene bodies of repressed genes.^81^ These genomic regions are characterized by different linker DNA and NFRs. For example, promoter regions located upstream of the TSS usually contain NFRs and have nucleosomes with more dynamic and disordered positioning. On the other hand, in gene bodies, nucleosomes are better positioned, especially close to the TSS.^82^ Thus, our results indicate that compaction behavior depends on whether macroH2A1 binds to gene bodies, promoters, or TSS.

It has been suggested that macroH2A1 levels are correlated with the progression of HD: mice models have indicated increased levels of macroH2A1 in neural regions as animals age and die.^14^ By studying the HD–like systems with a combination of 50% macroH2A1 and 50% canonical cores, we can mimic HD disease progression. Our results show that whatever effect macroH2A1 has on the chromatin fiber, either increasing or decreasing compaction, the strength of this effect is correlated with the level of macroH2A1. Namely, as we increase the level of macroH2A1 from 0 to 100%, there is a progressive change in chromatin compaction. These results support the idea that macroH2A1 levels could be used to detect the progression and severity of the disease. The resulting chromatin condensation may be correlated to the increase of the mutant htt protein.

Our results on the effect of hybrid cores further highlight how the substitution of one H2A copy by macroH2A1 instead of both can produce different results on genome folding and compaction. Thus, heterogeneous and asymmetric cores might offer an epigenetic mechanism of gene regulation during disease development and progression. Indeed, nucleosome asymmetry induced by differentially modified histones or a combination of histone variants has been proposed as a novel mechanism to regulate gene transcription.^83^ Additionally, mutated histones typically found in cancer are incorporated into heterogeneous nucleosomes, which might contribute to the disruption of genome architecture.^84,85^

While our model system incorporating only the histone fold domain of macroH2A1 provided some intriguing insights into structural alterations, a next step would involve incorporating the C-terminal domain of macroH2A1 and determining its influence on chromatin structure. The interactions of the C-terminal domain with other protein partners will be important to examine. Additional modeling and experimental studies that include both this domain and accompanying proteins will be essential to dissect full structural biophysics features of the HD genome.

Even though macroH2A1 appears to be a biomarker of HD, it may only be partly responsible for the manifestations of HD in patients. Other epigenetic factors such as methylation and acetylation likely work in tandem with the histone variant to favor HD development. Future clinical studies are required to further interrogate the relationship between macroH2A1 and HD.

Our analysis of the HTT locus reveals local rearrangements of 3D chromatin architecture which likely contribute to local dysregulation of transcription of the htt protein.^6^ In HD, there is both a loss of function of the normal htt protein and a gain of function of the mutated htt protein. The degree of the loss of expression of the normal htt protein depends on the number of CAG repeats; the more repeats, the more htt expression is reduced.^6^ Thus, it seems that the CAG repeats favor an epigenetic landscape that activates the transcription of the mutated HTT gene.

## Conclusions

Overall, our first-order HD–model helps explain how epigenetic factors like histone variants can regulate the folding and compaction of genes, which might influence the development of diseases. A clearer understanding of genome architecture can help develop new therapeutic avenues to diagnose and treat HD, including alternative genomic tools, like CRISPR-based tools.^86^ CRISPR has been used to delete the mutated region of the HTT gene, showing promising results in mice models.^80,87^ A combination of computational and experimental approaches on this problem will be fruitful.

Although we had not incorporated sequence effects in our model, we were able to fold the HTT gene system of size 150 kb (688 nucleosomes). The extension of CAG repeats was modeled by increasing the linker DNA length but it is not known if the CAG expansion occurs on nucleosomal or linker DNA; additionally, there are no nucleosome position data available for cell models of HD. Thus, it would be interesting to explore further aspects of changes associated with HD with a sequence-dependent resolution model on the atomic level to complement the chromatin kb viewpoint. Further modeling and experimental studies on HD will help to better diagnose and treat this devastating disease.

## Data Availability

Analysis scripts and representative structures for the Short NRL, Medium NRL, Medium NRL + NFR, and HTT systems are deposited in pdb format on the Zenodo repository (https://doi.org/10.5281/zenodo.15283868). The executable file of our chromatin Monte Carlo simulation code, together with input and output files, can be found at https://github.com/Schlicklab/Hi-BDiSCO.

## Supporting Information Available

Additional details on system specifications and analysis; convergence plots for the HD-like system of medium NRL and 100% macroH2A1 cores (Figure S1); global compaction plots for the HD-like systems with 100% canonical, 50% canonical, and 50% macroH2A1 cores, and 100% macroH2A1 cores (Figure S2); ratio of tail interactions for the HD-like systems with canonical and macroH2A1 cores (Figure S3); persistence length for the HD-like systems with canonical and macroH2A1 cores and packing ratio for the 61 bp linker DNA system with canonical and macroH2A1 cores (Figure S4); packing ratio for the HD-like systems with canonical, 50/50, and macroH2A1 cores with macroH2A1 cores uniformly distributed (Figure S5); persistence length and end-to-end distance for the HD-like systems with canonical, hybrid, and macroH2A1 cores (Figure S6); HTT gene chromatin configurations in healthy and diseased states (Figure S7); comparison between our HTT gene folds and published models for a 2 Mb genome region containing the HTT gene (Figure S8); position and value of the 300 charges calculated with DiSCO for the canonical, hybrid, and macroH2A1 cores (Table S1); position along the chromatin fiber of macroH2A1 and canonical cores in fibers random distribution of 50% canonical and 50% macroH2A1 cores (Table S2); and list of linker DNA lengths of the HTT gene in healthy and diseased conditions (Table S3) (PDF)
