# Supplementary material for: Regulation of Genome Architecture in Huntington’s Disease
Source: Biochemistry. 2025 Apr 27;64(9):2100–15. doi: 10.1021/acs.biochem.5c00029 (PMC12060273; doi:10.1021/acs.biochem.5c00029)
Supplement: Supplementary file 1 — bi5c00029_si_001.pdf [file bi5c00029_si_001.pdf]

Supporting Information
Regulation of Genome Architecture in Huntington's Disease

Stephanie Portillo-Ledesma[1,4], Minna Hang[1], and Tamar Schlick[1,2,3,4*]

[1]Department of Chemistry, 100 Washington Square East, Silver Building, New York University, New York, NY 10003 U.S.A.
[2]Courant Institute of Mathematical Sciences, New York University, 251 Mercer St., New York, NY 10012 U.S.A.
[3]New York University-East China Normal University Center for Computational Chemistry, New York University Shanghai, Shanghai 200122 China.
[4]Simons Center for Computational Physical Chemistry, 24 Waverly Place, Silver Building, New York University, New York, NY 10003 U.S.A.

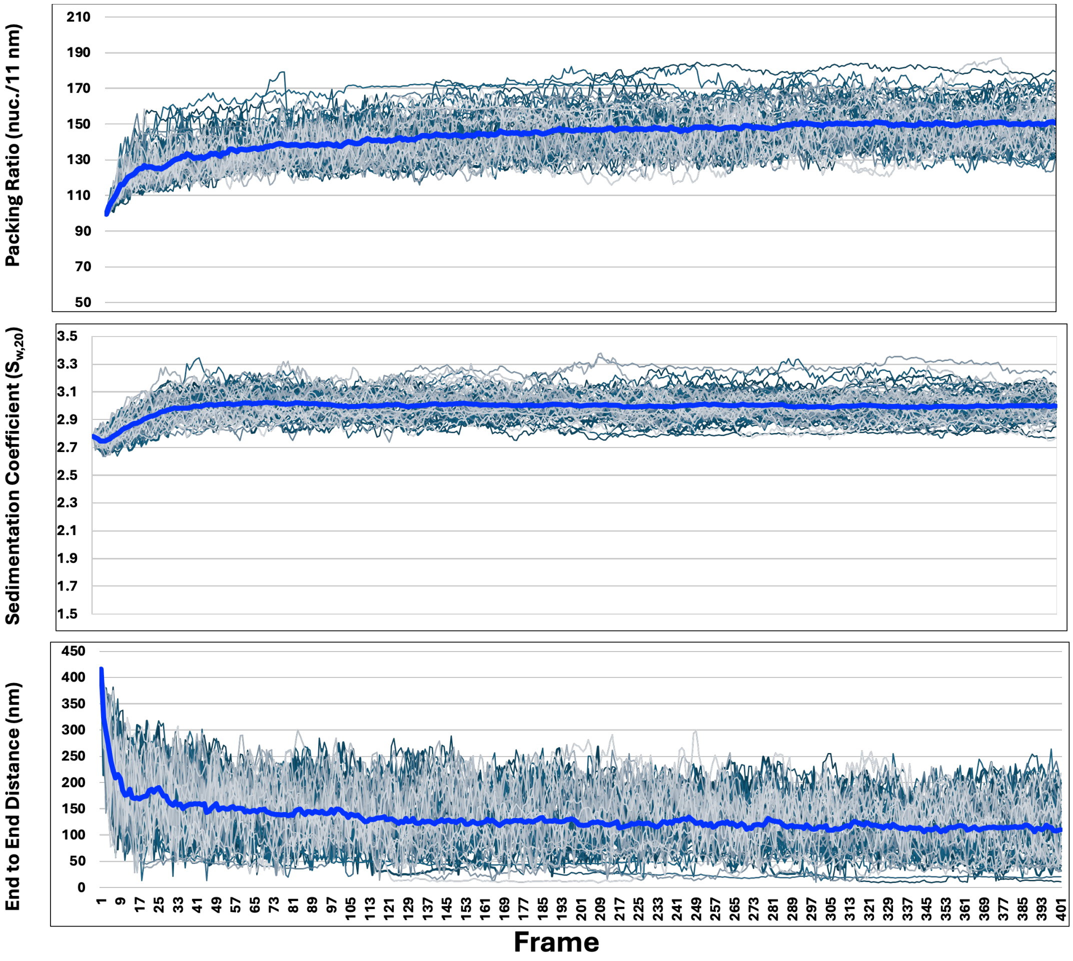

Figure S1. Example of convergence check. Plots of packing ratio, sedimentation coefficient, and end to end distance across each of the 60 trajectories for the medium NRL system with macroH2A1 cores. Tick blue line shows the average obtained from the 60 trajectories at each frame.

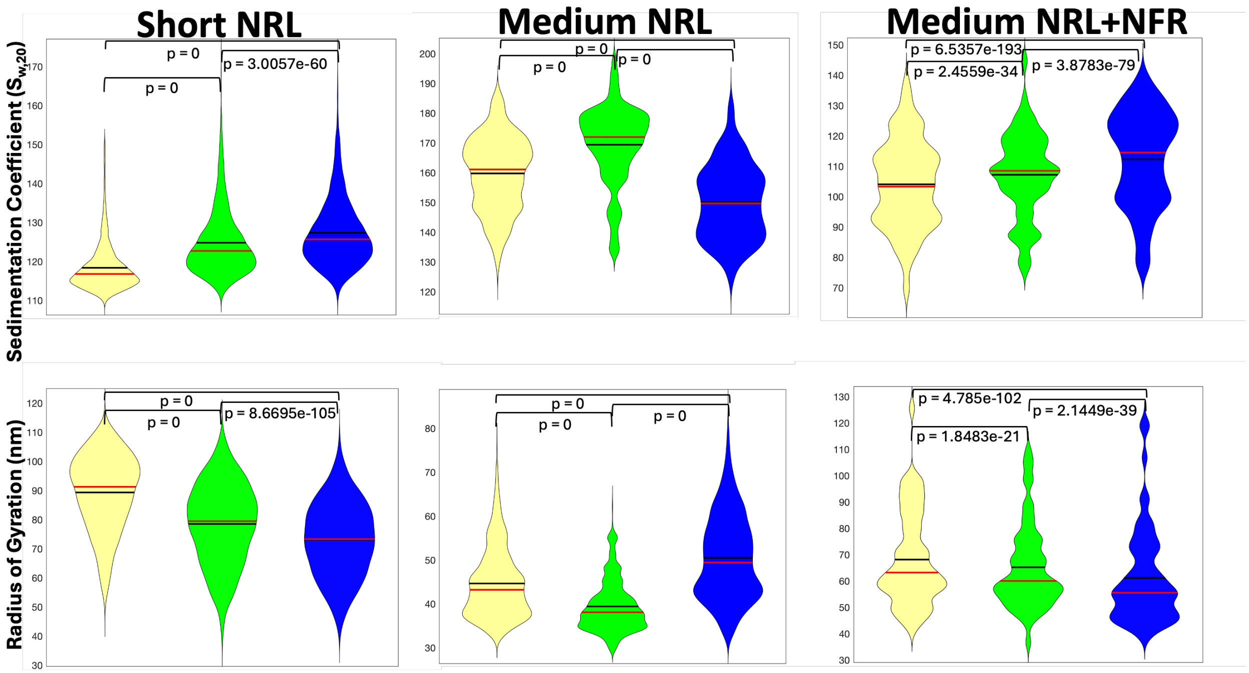

Figure S2. Global compaction properties: sedimentation coefficient and radius of gyration for the short NRL, Medium NRL, and Medium NRL + NFR systems with 100% canonical (yellow violins), 50% canonical and 50% macroH2A1 (green violins), and 100% macroH2A1 (blue violins) cores.

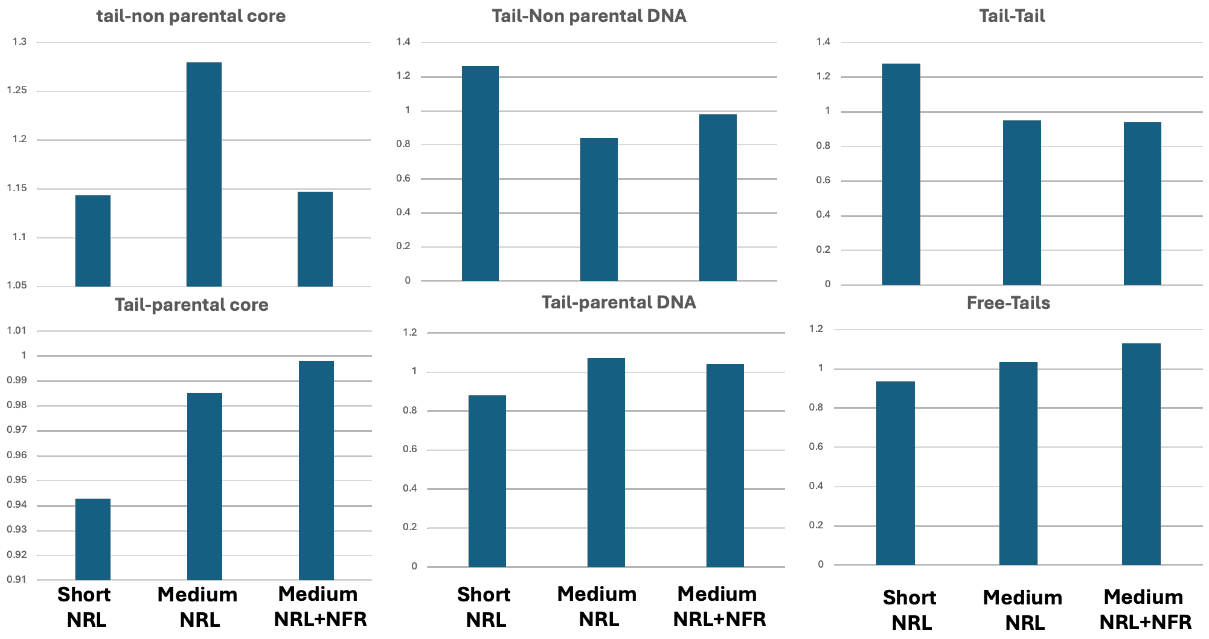

Figure S3. Ratio of total tail interactions between fibers with 100% canonical and 100% macroH2A1 cores. We measure the frequency of total tail interactions (H2A N-terminal, H2A C-terminal, H2B, H3, and H4) with different chromatin elements (parental cores and DNA, non-parental cores and DNA, other tails, or free tails) for every system studied (Short NRL, Medium NRL, Medium NRL+NFR).

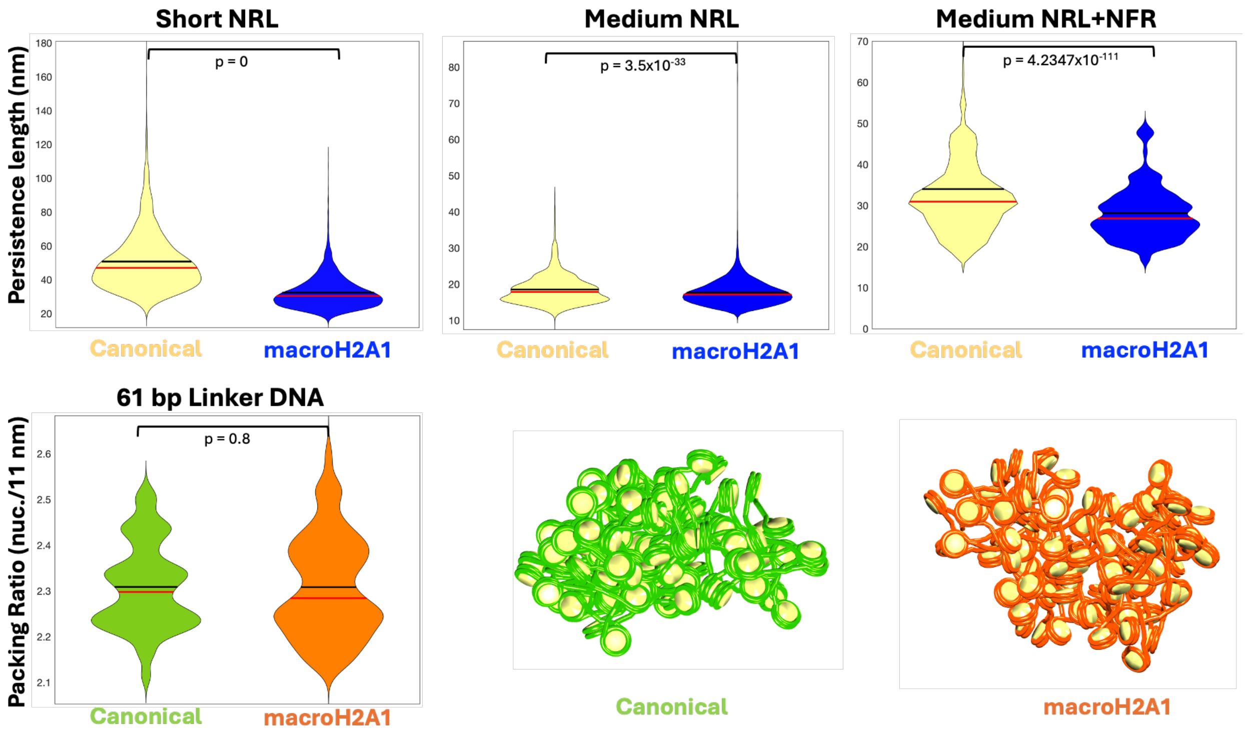

Figure S4. Top: Persistence length for the three HD-like systems with canonical and macroH2A1 cores. Bottom: Packing ratio for the 61 bp linker DNA system calculated from an ensemble of 5000 configurations, and one representative configuration for each system.

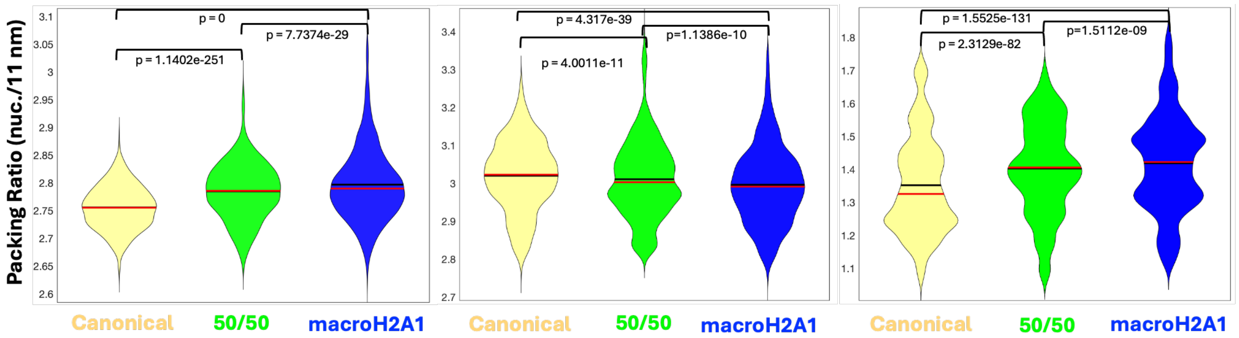

Figure S5. Fibers with 50% macroH1A1 cores and 50% canonical cores (green violins) uniformly distributed show an intermediate packing ratio that lays in between the systems with 100% of canonical (yellow violins) and 100% macroH2A1 cores (blue violins).

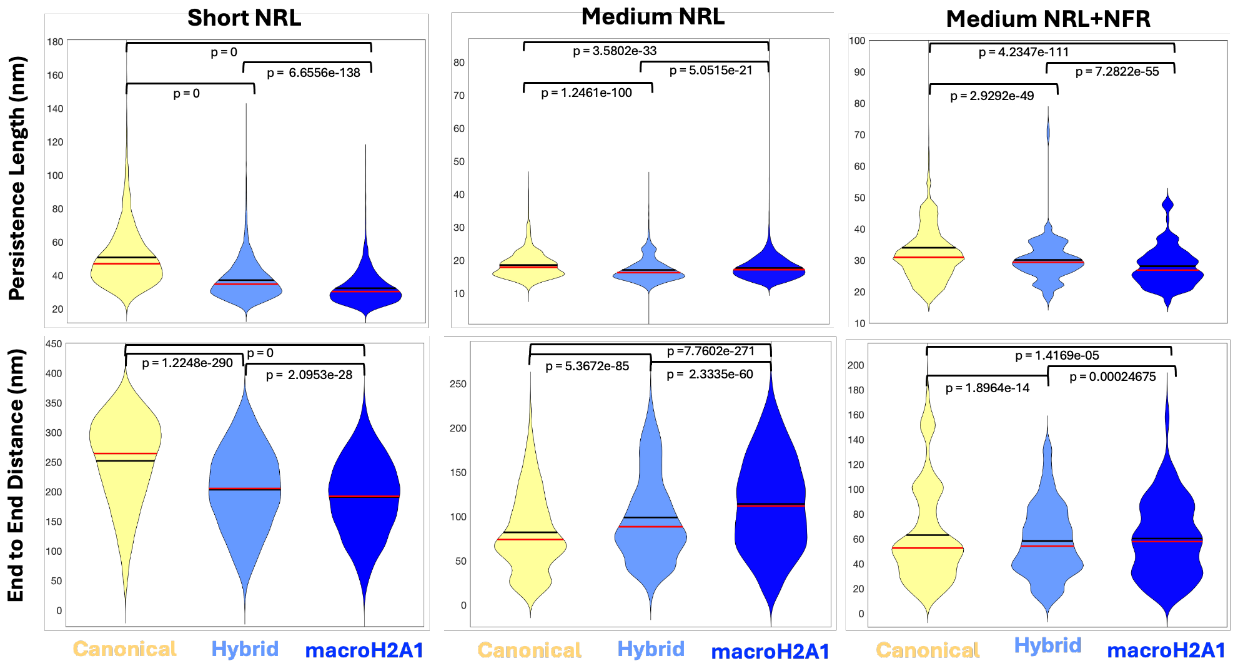

Figure S6. Persistence length and end-to-end distance for the Short NRL, Medium NRL, and Medium NRL+ NFR systems with 100% canonical (yellow violins), 100% hybrid (light blue violins), and 100% macroH2A1 (blue violins) cores.

Healthy

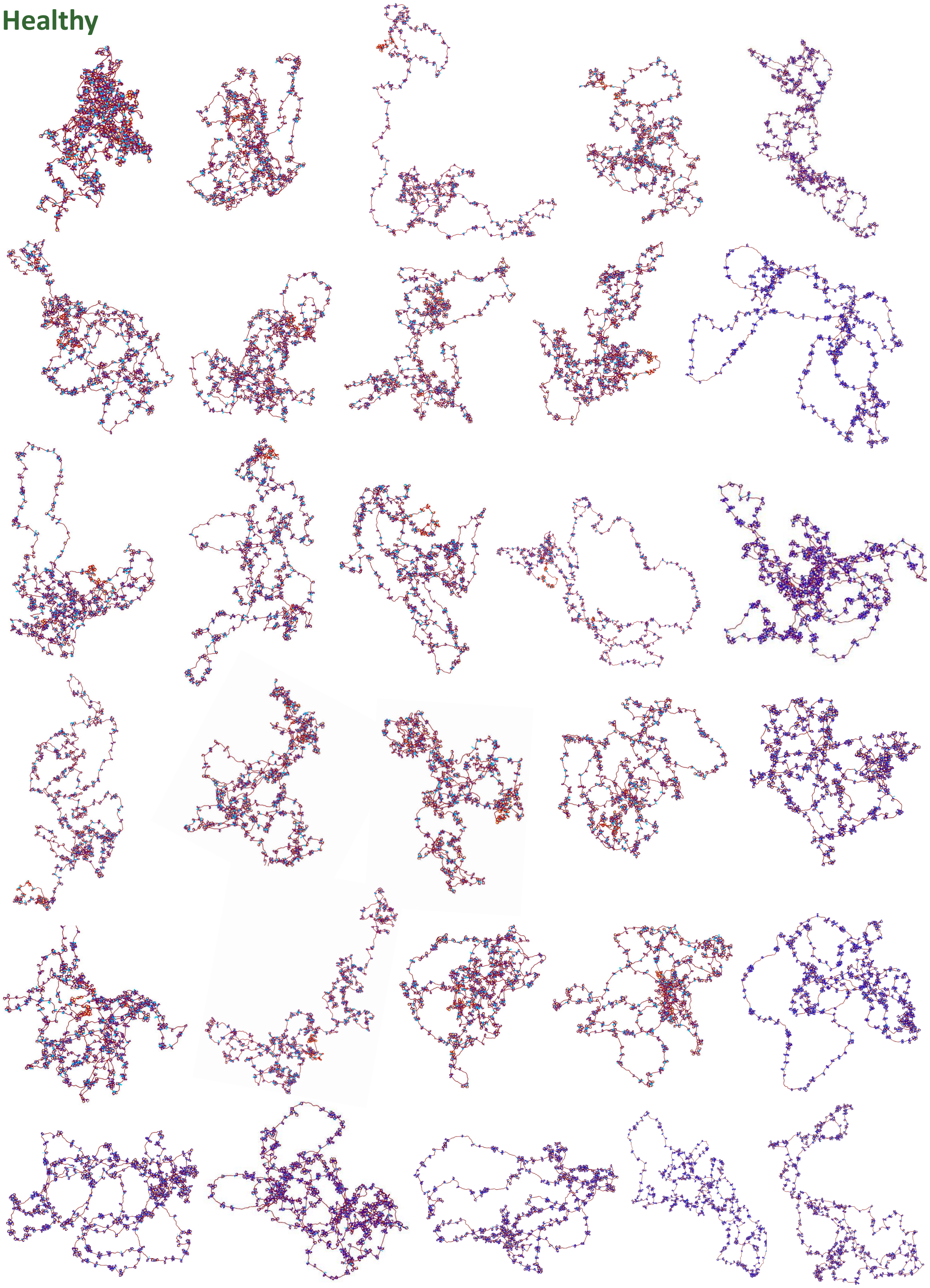

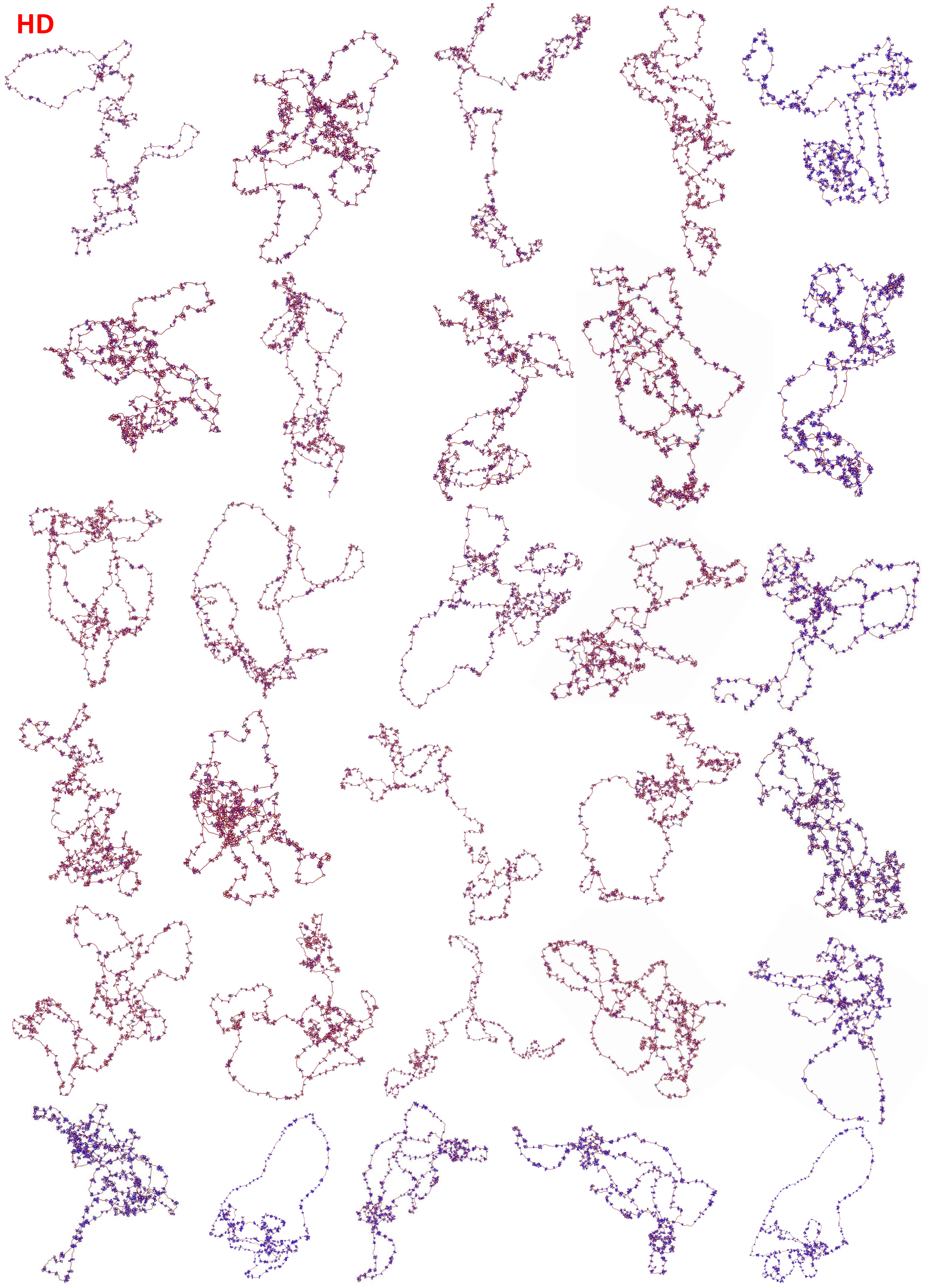

Figure S7. Final chromatin configuration for the 30 trajectories of the HTT gene in healthy and diseased conditions.

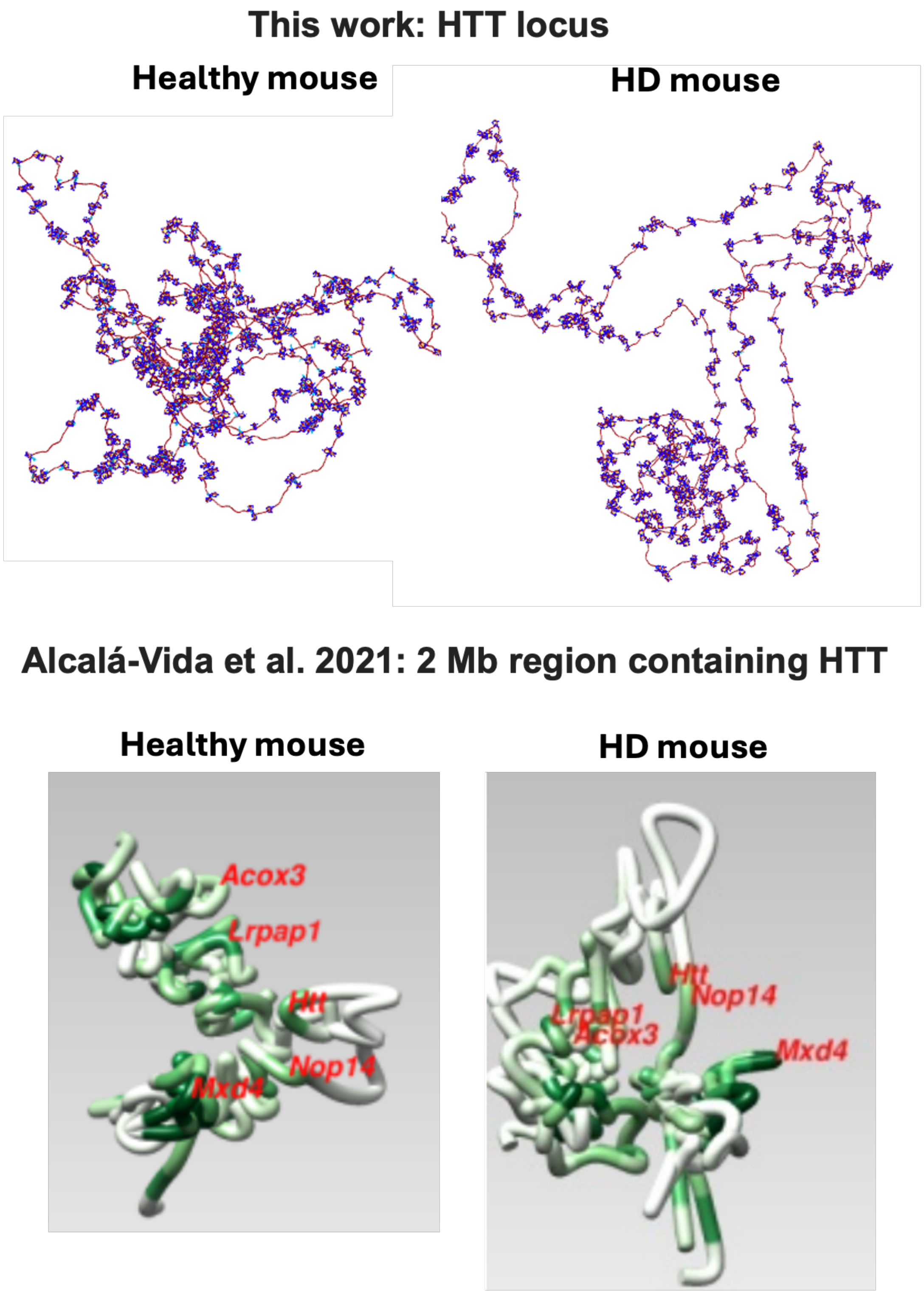

Figure S8. Comparison of our HTT healthy and diseased model gene conformations (top) with images from a published 2 Mb model (bottom) obtained from 4C-seq data of healthy and HD female mouse models are adapted from Alcalá-Vida et al. Nature Commun. 12:364 2021. Copyright 2021, The Author(s), licensed under a Creative Commons Attribution 4.0 License.

Table S1. Positions and values for the 300 charges calculated with the DiSCO algorithm for each nucleosome core: canonical, macroH2A1, and hybrid cores.

Canonical Core				macroH2A1 Core				Hybrid Core			
x	y	z	q	x	y	z	q	x	y	z	q
6.75	-1.66	19.41	-0.92	5.49	-5.18	16.71	-1.14	4.55	-4.86	8.37	-0.36
1.18	7.23	28.37	-1.39	-1.83	3.94	28.38	-0.26	-0.54	1.53	26.71	-0.40
-4.95	-5.05	20.09	-2.03	9.98	5.77	20.92	-3.71	10.04	1.83	17.82	-1.62
12.27	8.13	21.59	-2.53	-6.98	-11.52	25.21	-1.14	-0.15	-13.29	21.04	-0.18
-13.67	-7.42	28.63	-0.06	-6.52	-0.74	18.31	0.51	-4.39	-2.96	15.99	-0.21
3.31	-11.56	13.39	2.31	-0.29	-14.09	17.66	-0.48	6.48	10.55	23.86	-3.43
-11.06	6.19	23.66	4.71	-2.61	17.61	30.41	0.24	-14.28	-6.68	29.19	-1.06
11.58	-2.65	8.57	-0.63	-16.03	-5.65	27.67	-0.55	-4.00	17.74	31.79	0.54
-5.11	19.57	29.03	1.50	21.18	10.24	25.05	-0.47	-7.68	9.85	24.93	-1.10
-14.11	-2.39	21.05	-2.46	10.82	19.04	27.46	-2.53	18.82	16.97	29.66	0.55
-7.99	-17.30	21.79	-1.75	-12.00	9.05	24.28	1.29	10.56	17.88	26.58	-2.14
13.39	23.77	28.79	-0.76	-3.91	-24.38	25.50	-1.16	-11.08	-16.29	22.76	-1.95
3.03	16.61	19.72	-5.03	17.94	3.39	14.81	0.24	21.25	7.81	20.38	-0.43
-14.20	15.41	26.20	-0.03	2.53	16.31	20.66	-3.32	18.02	13.82	23.32	-0.26
24.13	12.06	22.10	2.82	-14.98	22.04	32.83	0.17	-13.51	23.22	33.21	0.13
3.22	25.85	25.38	-2.39	-15.89	-14.47	23.01	-2.21	-15.98	2.07	21.23	5.03
-15.10	26.62	31.13	-0.65	10.96	-16.30	13.45	-0.38	-4.05	-25.94	23.55	-1.16
-25.35	-4.15	25.91	0.31	20.76	24.43	28.48	0.31	11.72	-16.49	13.20	1.23
0.29	-23.72	18.87	-0.74	-6.04	28.18	29.59	1.23	0.69	20.37	21.60	1.09
22.98	1.40	14.72	-1.70	-18.97	2.05	21.05	0.57	-14.85	16.96	25.66	-1.11
20.73	21.39	22.55	-1.84	3.97	-23.75	17.82	-0.19	-24.55	-6.90	25.15	-2.08
-8.76	26.60	24.60	5.27	15.46	-6.25	9.45	0.34	5.26	-24.32	17.47	-1.35
-26.90	9.47	25.47	-0.84	5.06	28.93	24.79	1.27	18.92	-4.36	10.06	1.84
-21.55	3.63	20.07	-0.65	-17.76	17.70	24.13	-2.17	16.28	27.69	26.09	-1.36
-24.86	-22.98	26.93	-1.59	-27.91	9.73	26.70	-0.38	-8.57	35.02	31.47	0.98
10.62	-20.95	14.52	-1.55	-27.66	-23.68	28.68	-0.74	-27.25	10.33	26.31	-1.27
41.22	5.27	25.22	-0.45	-13.07	-30.68	24.16	-1.05	-27.59	-24.26	28.30	-0.85
38.12	17.81	26.43	0.12	42.07	1.56	24.60	-0.96	-12.97	-31.07	23.65	-1.11
17.97	29.80	23.80	-1.84	38.83	16.76	26.05	-1.10	43.52	3.93	25.74	-0.92
-25.43	21.44	25.24	-2.19	25.13	19.16	19.92	0.22	37.59	18.68	26.10	-0.29
-16.86	12.94	17.14	-1.57	-17.17	33.36	28.26	0.28	27.91	18.28	20.90	-1.26
-39.00	3.42	28.78	-0.82	-30.54	21.31	28.32	-0.63	-19.46	31.53	28.12	0.73
-36.08	-14.65	27.64	-0.79	-37.14	8.34	28.48	-0.82	-29.53	23.03	28.46	-0.70
-12.49	-32.91	22.58	-1.71	-32.69	-7.81	24.42	-1.55	-20.57	10.80	18.74	0.98
0.12	-34.78	21.15	-0.65	-26.02	-15.12	21.75	-0.82	-26.75	1.79	20.41	0.48

25.15	-10.35	14.64	0.00	-20.17	-23.54	20.43	-0.72	-36.91	-13.68	27.49	-1.43
34.72	-0.50	17.30	-0.33	2.26	-38.70	22.60	-1.22	-21.09	-20.94	19.37	-0.84
34.13	10.53	19.05	-2.94	30.73	-9.24	17.04	-1.01	-10.51	-40.66	25.20	-0.79
30.03	18.97	19.73	-1.83	25.72	-2.48	11.83	-0.67	2.79	-38.31	21.61	-1.05
9.52	30.05	19.32	3.31	33.03	8.68	17.33	-2.26	33.23	-1.85	15.47	-1.26
-22.61	32.47	25.03	-3.12	48.28	12.19	25.95	-0.62	36.98	10.09	20.14	-3.44
-35.33	18.41	25.30	-1.47	26.95	29.54	22.32	-2.01	23.16	26.07	20.33	0.45
-32.07	2.16	21.32	-0.70	15.70	34.07	21.95	-3.42	0.30	33.60	21.05	-0.97
-33.52	-8.17	21.71	-2.66	-5.84	37.13	23.58	-2.10	-18.49	24.91	20.80	-1.07
-26.67	-15.91	19.23	-3.31	-22.04	26.58	22.51	-1.43	-29.22	17.16	22.65	-1.12
-17.41	-23.89	17.59	2.49	-33.01	4.06	21.76	0.09	-39.76	8.60	25.56	-2.23
-9.28	-41.68	23.15	-1.18	-26.64	-3.51	17.17	0.04	-35.02	-4.86	21.70	-0.55
6.16	-30.01	15.19	0.05	-38.19	-18.58	25.24	-1.71	-29.52	-14.64	19.36	-2.04
51.47	-12.74	27.50	-0.08	-29.36	-32.49	25.13	-1.61	-29.29	-36.61	25.74	-1.18
40.71	-10.46	21.15	0.11	-10.31	-40.99	22.73	-1.41	-4.98	-34.00	16.98	-2.14
49.18	10.06	22.90	-1.31	-4.65	-31.67	15.98	-0.19	5.56	-32.82	15.78	1.08
41.74	14.19	19.71	-2.56	16.10	-33.51	17.40	-1.40	17.79	-28.86	14.79	-2.28
38.26	29.46	22.49	-1.45	50.62	-13.46	26.19	0.19	51.01	-14.22	26.23	0.07
15.43	41.28	21.75	-0.85	41.65	-8.90	20.64	0.87	42.59	-7.02	20.84	0.03
-7.62	42.53	22.36	-0.34	41.06	25.28	22.88	-1.10	48.00	14.12	22.40	-1.06
-13.60	34.57	19.68	-3.39	32.91	23.68	18.37	-0.75	37.77	27.45	21.74	-2.67
-22.43	26.04	18.59	1.50	23.34	37.20	21.14	-0.60	22.14	35.40	23.79	-0.94
-40.03	10.19	21.88	-1.72	6.64	36.43	18.81	0.10	10.77	40.62	21.12	-1.43
-40.17	-21.89	22.93	-0.71	-26.90	37.03	24.32	-0.28	-7.63	43.31	22.97	-0.84
-27.54	-37.15	22.19	-1.38	-40.49	18.07	23.60	-0.98	-17.85	40.79	23.54	-0.79
-20.33	-32.50	18.17	1.87	-44.76	3.74	23.49	-1.06	-39.99	19.41	23.54	-1.17
4.80	-43.57	19.33	-1.87	-39.29	-3.13	20.54	-0.74	-45.46	-0.86	23.32	-0.13
17.84	-32.56	15.46	-2.37	-31.58	-23.22	19.62	-0.26	-39.70	-23.65	22.35	-1.84
18.50	-9.38	7.81	2.83	-21.02	-33.59	18.23	0.18	-22.63	-31.92	17.65	-2.91
49.72	-3.35	21.81	-0.72	-4.11	-40.15	17.26	-1.39	-4.21	-45.02	19.02	-0.95
59.05	0.35	21.78	-1.16	5.95	-33.60	13.57	0.27	8.29	-46.51	19.36	-0.93
49.60	20.97	20.30	-0.75	23.42	-22.92	12.11	-0.50	33.17	-17.07	14.06	-0.92
27.64	38.60	18.80	-1.26	34.90	-20.79	15.98	0.43	24.49	-13.20	9.43	-2.69
6.13	39.23	16.54	-0.58	50.72	-3.95	21.94	-2.67	59.31	-1.36	21.00	-1.45
-2.42	33.30	17.14	-2.27	50.36	5.76	18.27	-1.29	49.26	0.77	17.03	-3.52
-21.41	41.05	20.63	0.10	50.56	20.04	20.02	-0.49	43.69	21.30	17.80	0.28
-32.62	28.61	19.00	0.81	38.93	33.66	19.65	-0.92	29.96	33.48	17.34	-1.86
-42.39	17.74	20.62	-1.18	13.36	46.84	19.92	-1.01	-0.29	41.63	17.91	-1.13
-45.57	-2.19	19.51	-1.56	-10.75	44.41	20.24	-1.35	-13.90	46.59	20.84	-0.60
-31.65	-27.22	17.47	-4.07	-19.72	40.90	19.72	-2.14	-29.63	33.89	19.66	-1.44

-15.69	-40.45	17.10	0.28	-32.14	25.30	18.41	-0.54	-33.37	24.95	18.28	0.45
-4.26	-47.25	17.71	-1.98	-39.63	10.31	17.90	-1.81	-38.36	-12.41	17.07	0.33
15.41	-26.36	10.83	2.75	-38.95	-12.86	17.08	-1.65	-32.96	-30.71	18.09	-0.98
49.68	-12.62	18.67	-0.01	-39.67	-23.69	19.15	-0.90	-16.41	-40.94	16.25	3.31
35.02	-9.50	13.03	-0.11	-29.25	-41.22	19.46	-1.48	-12.01	-47.66	18.29	-2.30
49.73	3.05	14.49	-2.40	-7.61	-50.61	18.59	-0.63	21.52	-38.80	14.96	-1.30
44.69	26.69	16.33	-0.78	8.93	-47.62	16.83	-0.94	30.33	-29.14	13.22	-1.56
32.20	30.33	13.66	3.14	26.31	-36.95	15.04	-0.66	15.73	-12.40	4.65	2.38
10.54	47.32	16.99	-0.85	18.89	-15.56	6.52	0.68	56.62	-10.16	19.99	-0.59
-3.86	41.47	14.73	-0.93	56.86	-10.53	20.35	-0.99	49.99	9.72	14.32	0.54
-14.40	47.97	17.06	-1.28	60.34	-0.06	17.61	-1.23	52.37	19.13	16.19	-1.32
-32.03	39.51	17.57	-1.22	44.58	24.24	15.34	-1.71	21.20	47.38	16.58	-1.29
-36.66	21.60	14.91	-1.46	30.45	34.73	14.20	0.24	6.23	50.06	16.62	-1.12
-38.76	-14.79	14.44	-1.79	23.13	46.43	16.53	-0.83	-6.60	45.79	15.62	-1.02
-32.84	-35.42	15.45	-1.49	-1.10	43.65	14.68	-2.22	-25.09	42.27	16.87	-1.82
-24.23	-39.40	14.48	-1.69	-32.85	38.79	17.58	-1.33	-44.74	17.93	16.46	-1.44
10.02	-38.98	11.38	1.02	-40.53	22.82	16.16	-1.78	-44.52	7.13	15.74	-1.71
26.78	-37.92	13.07	-0.93	-44.23	3.63	15.17	-1.70	-42.92	-4.86	14.28	-5.10
30.27	-25.88	10.84	-1.47	-43.65	-6.70	14.82	-1.01	-27.98	-41.88	15.34	-2.00
22.74	-19.30	7.59	1.52	-31.13	-32.12	14.54	-2.29	1.73	-48.35	13.09	-1.00
57.28	-7.62	16.88	-1.58	-18.41	-43.07	13.85	-1.24	13.01	-42.38	11.42	-0.02
49.61	13.98	11.63	-2.53	-0.26	-47.68	13.40	-1.16	26.19	-22.42	7.95	4.22
40.23	33.17	12.70	-1.99	12.87	-40.45	11.25	-0.59	47.81	-13.80	13.74	-0.01
21.62	46.79	12.64	-1.94	32.63	-29.92	11.50	-1.24	57.50	2.83	12.80	-0.77
2.50	48.32	12.27	-0.66	38.97	-11.97	10.55	0.49	46.50	24.83	11.62	-1.34
-21.40	45.12	13.03	-2.24	49.92	13.85	12.01	-3.25	41.23	35.30	13.04	-1.16
-36.74	33.28	13.07	-2.13	41.00	30.78	11.63	-1.27	24.74	39.60	10.96	-2.02
-45.68	7.96	12.16	-1.65	24.10	39.89	11.05	-2.42	0.52	47.01	11.76	0.70
-39.87	-26.21	12.21	-0.76	6.62	52.04	13.36	-0.89	-19.67	49.38	13.60	-1.23
-18.76	-41.51	11.16	-2.23	-16.67	48.83	13.65	-1.69	-37.03	35.99	13.34	-1.17
5.65	-51.19	12.30	-1.49	-25.83	42.36	12.92	-1.12	-37.78	20.97	11.18	-0.49
14.69	-47.90	11.02	-0.39	-46.52	15.28	12.73	-1.49	-39.52	-22.52	10.71	-3.29
23.14	-30.88	7.95	-0.23	-47.91	-1.53	12.22	-0.79	-31.02	-34.80	10.49	-0.51
28.93	-11.72	6.09	-2.06	-39.13	-28.65	11.63	-1.58	-7.25	-49.88	10.51	-1.54
44.50	-11.52	10.59	-0.63	-10.81	-46.92	11.05	-2.04	23.17	-32.19	7.82	1.84
60.64	1.06	9.79	-0.79	6.59	-52.05	11.10	-0.90	30.85	-38.66	9.57	-0.59
49.89	23.50	8.83	-0.80	20.92	-40.85	9.84	-1.10	39.69	-12.07	8.15	4.42
27.81	38.90	7.98	-2.96	50.22	-13.64	11.34	0.22	54.76	-6.36	11.37	-2.52
9.89	53.45	9.76	-1.40	57.35	-3.70	8.39	-2.73	47.26	15.76	7.19	-1.39
-10.75	44.74	8.28	-0.81	56.52	6.62	8.43	-0.77	39.29	28.11	7.26	-1.66

-28.44	40.22	9.00	-0.78	36.81	38.42	9.01	-1.04	14.76	49.68	9.79	-1.46
-36.68	26.63	8.67	0.82	16.16	49.40	8.72	-1.78	7.66	46.33	7.85	-1.20
-43.83	-5.65	7.93	-0.30	-8.39	48.38	9.20	0.60	-13.29	43.10	7.83	-1.66
-28.82	-36.30	7.74	-2.64	-17.23	41.57	8.00	0.11	-28.45	38.54	8.43	-2.67
-9.85	-47.85	7.78	-2.62	-33.76	31.42	8.77	-2.74	-42.87	12.96	7.77	-0.24
20.10	-40.83	6.76	-2.08	-39.92	18.90	8.24	1.62	-41.57	-12.36	7.68	1.39
36.22	-33.70	7.78	-0.65	-40.06	-18.59	8.00	-1.26	-21.21	-44.17	7.17	-2.19
54.73	-13.69	9.08	0.16	-27.62	-40.18	7.65	-2.45	9.66	-50.61	7.01	-1.38
52.74	-4.06	8.82	-1.71	-3.78	-53.28	7.92	-0.71	39.12	-29.71	6.66	-1.06
52.45	6.18	4.68	0.05	32.21	-39.88	7.08	-1.53	57.04	-15.76	8.87	0.28
40.89	26.71	5.05	-2.07	33.04	-20.80	4.92	-0.57	47.35	-10.89	6.47	-2.84
35.71	34.97	4.69	-0.78	59.18	-11.61	11.54	0.36	51.03	5.52	3.85	-2.83
19.15	42.02	4.47	-3.13	44.94	20.58	4.62	-1.77	36.56	36.33	4.71	-0.32
-3.31	49.59	5.06	-1.78	29.55	37.87	4.21	-0.83	20.61	44.76	4.70	-0.06
-19.92	40.94	4.74	-2.54	10.52	45.09	4.29	-0.04	1.86	51.68	5.49	-0.93
-40.80	16.21	5.28	-1.23	1.03	49.86	4.77	-1.88	-24.93	47.33	5.57	-0.61
-40.50	-18.51	4.44	-1.22	-23.87	47.14	5.25	-0.84	-37.11	30.37	5.14	-0.44
-0.65	-49.24	4.91	-0.70	-27.78	38.33	4.70	-1.18	-44.02	1.46	4.69	-1.87
30.45	-41.73	4.43	-1.08	-44.05	7.68	4.36	-1.17	-36.12	-29.36	3.85	-1.32
36.05	-19.68	3.30	-0.68	-33.07	-32.29	4.63	1.18	0.24	-53.01	3.95	-1.28
40.71	-13.44	3.95	0.30	13.19	-48.02	4.22	-2.58	18.41	-39.93	3.25	-5.67
46.95	16.51	1.68	-0.69	39.49	-29.59	4.44	-0.98	34.59	-21.30	2.46	1.18
24.92	38.89	1.12	0.92	44.15	-15.01	3.41	-0.74	26.79	-16.14	1.48	1.17
6.54	47.86	1.27	-1.36	49.40	11.22	1.30	0.60	55.27	-3.86	0.91	-0.49
-13.34	43.09	1.89	0.89	37.43	30.82	1.36	0.34	26.82	37.43	1.08	-1.37
-32.15	33.10	1.59	-0.89	19.60	42.83	0.81	-0.44	12.29	43.46	0.55	-1.85
-48.79	3.25	1.35	-0.14	-10.05	43.67	0.84	-1.62	-4.81	44.28	0.59	-2.06
-35.55	-29.07	1.71	2.43	-37.38	25.67	0.89	-0.03	-40.84	21.11	0.67	-1.42
9.56	-48.96	1.60	-2.40	-43.26	-7.55	1.38	-0.41	-40.38	-19.84	1.07	0.16
16.99	-45.84	1.06	-1.33	-18.05	-44.78	1.24	-0.59	-12.63	-47.85	0.97	-0.32
42.12	-27.85	1.52	-1.56	15.33	-29.69	0.07	-0.56	12.60	-45.84	0.31	-2.51
49.69	-9.81	1.11	-2.75	25.12	-34.90	0.39	-1.87	14.42	-28.31	-0.26	0.93
49.83	-0.65	-1.66	-1.02	54.41	-10.94	1.11	-0.99	31.83	-33.76	0.24	-7.25
16.27	49.54	-1.88	-0.86	53.79	0.74	-2.63	-0.31	41.91	-16.34	0.21	-0.65
-5.82	45.37	-2.23	-0.86	42.37	24.99	-1.99	-1.55	57.23	-11.76	1.17	-0.39
-41.36	26.01	-2.39	-1.04	-19.78	39.89	-2.40	0.38	42.82	21.94	-2.28	-0.67
-43.76	11.54	-2.25	-0.60	-46.87	19.77	-2.88	-0.67	-19.25	40.23	-2.93	-0.46
-48.37	-9.82	-2.35	-1.41	-46.41	0.96	-2.69	-1.15	-30.06	35.25	-3.00	-1.07
-48.77	-21.40	-2.46	-0.50	-45.27	-18.53	-2.72	-0.76	-43.66	8.96	-2.90	-0.51
-22.52	-44.40	-2.23	-0.26	3.23	-52.20	-2.59	-1.31	-45.56	-7.86	-2.91	-0.95

22.23	-35.23	-1.81	-1.72	13.38	-55.81	-2.74	-0.14	-28.67	-38.45	-2.81	-0.52
34.22	-34.64	-2.04	-2.04	34.96	-38.27	-2.50	-1.04	10.21	-55.84	-2.95	-0.88
42.82	-19.38	-1.98	-1.11	44.67	-25.71	-2.49	-2.07	22.47	-33.12	-2.33	-2.63
43.10	24.21	-5.51	-0.76	46.60	17.19	-5.29	-0.40	43.11	-29.21	-2.75	-1.08
32.40	34.71	-5.46	-0.98	3.61	44.79	-6.03	-1.26	48.78	-10.82	-2.34	-0.92
-24.42	37.84	-5.73	-2.11	-35.64	37.44	-6.97	-0.82	47.74	11.90	-5.52	-1.28
-47.02	19.52	-6.36	-0.91	-37.60	29.23	-6.55	-1.78	35.55	30.92	-5.55	-0.52
-49.61	-1.41	-6.14	-2.29	-44.00	10.30	-5.92	-0.26	-11.12	44.92	-6.61	-0.01
-41.29	-23.05	-4.99	-2.07	-37.05	-26.98	-4.82	-0.97	-38.35	29.12	-6.89	-2.40
-33.05	-38.20	-5.88	-0.68	-33.11	-39.89	-6.38	-0.49	-49.13	1.17	-6.83	-1.09
-5.06	-55.54	-6.12	-0.57	-8.66	-48.98	-5.29	-1.08	-48.22	-18.13	-6.31	-1.31
12.94	-57.20	-6.29	-0.27	16.70	-40.99	-4.51	-1.98	-21.86	-47.82	-6.48	-0.82
12.90	-29.59	-3.47	-1.24	16.65	-22.23	-3.20	-0.04	-4.38	-56.79	-6.72	-1.01
29.38	-40.14	-5.25	-0.67	32.80	-27.24	-4.74	-1.94	14.20	-38.05	-4.81	-0.60
47.72	-14.15	-5.26	-0.60	47.43	-13.36	-5.51	-1.49	35.12	-27.30	-4.23	-0.79
50.97	9.90	-9.10	-0.85	38.45	33.20	-9.76	-1.06	43.21	-21.81	-4.88	-2.00
10.12	42.10	-8.52	0.72	28.25	42.40	-10.07	-1.21	50.06	0.83	-8.73	-0.41
-14.79	44.30	-9.46	-1.22	-13.61	46.62	-10.38	-0.35	27.20	41.07	-9.78	-1.19
-36.82	35.44	-10.38	-1.45	-25.69	36.14	-9.77	-0.57	3.59	46.16	-10.11	-1.16
-44.74	5.66	-8.97	0.23	-50.29	16.41	-11.24	-0.34	-24.51	37.16	-9.77	0.00
-45.77	-15.77	-9.17	-1.08	-50.97	-9.09	-10.75	-1.39	-48.10	19.15	-7.98	-1.15
-35.71	-29.76	-8.31	-2.58	-44.83	-24.89	-10.38	-0.23	-47.58	10.92	-10.75	-0.26
-15.70	-49.50	-9.43	-0.74	-23.90	-49.42	-10.37	-0.62	-51.18	-7.86	-11.00	-1.10
4.50	-51.98	-6.92	-1.49	-1.01	-60.31	-10.89	-0.76	-37.35	-27.75	-8.95	-1.91
13.12	-39.62	-6.75	-0.33	7.77	-57.12	-10.29	-2.28	-11.55	-49.18	-9.51	0.86
21.83	-26.26	-5.69	-1.77	23.56	-28.14	-5.89	-1.13	15.80	-49.66	-8.80	0.49
41.66	-29.98	-8.69	-0.61	29.14	-34.87	-7.86	-0.69	31.66	-38.88	-8.51	0.07
49.51	0.53	-11.90	-2.32	49.71	-3.70	-11.64	-2.40	30.55	-21.32	-6.60	-1.64
39.96	32.20	-13.04	-1.43	51.40	8.28	-12.21	-1.99	52.47	-7.71	-9.62	-1.27
23.42	42.11	-12.74	-1.09	16.43	41.18	-11.41	-1.88	42.69	25.87	-13.32	-1.93
-0.45	44.89	-12.48	-1.96	-3.90	44.61	-12.90	-2.01	39.96	34.08	-14.23	-0.90
-42.94	27.16	-14.40	-2.26	-21.29	42.99	-14.28	-1.51	14.64	40.92	-12.17	-1.21
-48.36	13.22	-14.16	-2.19	-43.11	23.83	-13.94	-2.62	-17.99	43.89	-14.49	-1.22
-51.28	-8.02	-14.34	-0.73	-44.71	2.23	-13.31	-1.38	-34.04	34.91	-14.93	-1.36
-45.76	-24.46	-14.02	-0.66	-46.04	-16.51	-13.41	-2.38	-42.61	24.58	-14.99	-1.57
-25.13	-42.18	-12.42	-3.75	-34.51	-32.72	-13.02	-2.66	-42.97	-3.04	-13.01	-1.82
-7.47	-49.80	-12.54	-0.69	-26.05	-40.67	-12.84	-1.33	-34.37	-38.92	-13.60	-1.92
2.24	-59.37	-14.59	-0.74	-14.56	-50.77	-13.52	-1.45	-19.05	-52.02	-14.36	-1.37
15.31	-48.40	-11.57	0.50	15.98	-49.35	-11.94	1.20	5.85	-60.99	-15.21	-2.27
31.97	-24.12	-9.27	-3.39	19.93	-35.27	-9.33	-2.01	13.91	-57.60	-12.92	-0.19

43.91	-21.03	-11.45	-0.76	25.46	-17.52	-7.09	0.19	21.17	-24.64	-7.64	-0.73
50.43	-8.68	-12.18	-1.15	44.93	16.99	-15.65	-0.90	41.01	-12.72	-10.46	-1.48
43.71	17.26	-14.45	-3.52	42.94	27.26	-16.95	-1.99	50.82	9.95	-16.91	-1.57
30.86	35.99	-15.13	-1.14	6.73	42.04	-15.86	-1.54	43.00	16.11	-14.87	-1.74
-8.60	44.41	-16.41	-0.96	-14.83	45.53	-18.18	-0.90	30.24	34.79	-16.43	-0.73
-27.12	31.24	-15.51	-1.91	-35.67	33.25	-18.73	-1.35	-4.08	44.47	-16.93	-3.44
-40.82	18.36	-16.62	0.31	-30.54	26.23	-15.97	-0.73	-12.31	46.52	-18.65	-0.95
-38.04	0.04	-13.84	-0.37	-46.00	10.58	-17.39	-1.43	-29.57	24.54	-15.74	-0.51
-41.74	-8.85	-15.48	-1.74	-36.44	-6.20	-14.28	-1.17	-47.21	16.53	-19.32	-0.65
-40.78	-17.55	-15.81	-2.75	-39.36	-26.56	-16.98	-1.46	-45.27	-15.20	-17.20	-2.68
-31.69	-35.11	-16.12	-0.85	-20.52	-46.19	-16.88	-1.81	-42.04	-24.50	-17.14	-0.67
-19.44	-51.45	-18.02	-1.03	-0.33	-48.06	-15.32	-0.60	-25.67	-43.73	-17.22	-1.84
0.68	-50.87	-16.13	-4.17	12.28	-54.95	-17.66	-1.31	-0.26	-47.85	-15.29	-1.55
11.22	-55.23	-17.67	-0.48	14.71	-28.16	-9.42	-0.22	-0.58	-56.01	-17.94	0.06
13.78	-17.01	-6.09	2.85	40.05	-23.41	-14.02	-1.26	20.29	-36.84	-12.41	0.00
25.83	-15.09	-8.64	4.63	35.71	-8.76	-11.11	-0.37	13.86	-16.09	-5.95	0.64
38.22	-5.95	-11.68	-1.56	42.39	3.12	-16.41	-1.07	38.56	-24.96	-14.03	-1.05
48.35	8.32	-19.01	-1.05	35.18	35.12	-20.43	-0.76	46.46	-3.86	-18.08	-2.66
31.69	22.65	-15.74	2.45	23.55	41.05	-20.62	-1.52	32.66	21.89	-16.75	-1.17
14.06	38.64	-17.78	-3.06	-0.95	47.06	-21.63	-1.42	22.12	40.82	-20.39	-1.37
5.39	44.99	-19.87	-1.21	-25.27	34.35	-20.11	-1.53	5.90	40.55	-19.40	-1.54
-19.51	40.10	-20.30	-2.01	-40.76	26.27	-22.94	0.09	-24.11	34.78	-20.84	-2.02
-34.55	25.83	-19.92	-2.62	-39.30	15.35	-20.30	-3.95	-39.94	27.73	-22.97	-0.46
-40.27	8.48	-18.96	-2.56	-45.01	-8.18	-20.51	-0.39	-45.58	6.39	-19.67	-1.20
-34.33	-6.83	-16.06	0.62	-33.19	-17.99	-17.21	-1.49	-33.35	0.47	-16.61	-1.98
-40.67	-26.33	-19.78	-0.09	-31.95	-39.65	-21.56	-0.84	-33.64	-16.35	-17.30	-2.44
-31.14	-25.14	-17.30	-3.11	-7.90	-49.87	-20.14	-1.16	-28.02	-28.60	-17.50	-1.45
-27.97	-41.73	-20.82	-0.67	1.82	-57.30	-22.20	-1.88	-29.40	-36.71	-20.04	-0.56
4.77	-58.50	-22.51	-0.96	13.64	-39.96	-15.33	1.60	-9.89	-49.93	-20.37	-2.55
3.18	-32.58	-12.50	-0.95	13.97	-13.91	-6.41	-0.04	15.79	-49.59	-19.10	-0.48
13.42	-40.81	-15.57	-0.45	32.48	-17.33	-13.37	-1.60	12.88	-29.36	-11.70	0.88
36.36	-20.13	-14.80	-1.30	42.88	-7.05	-16.40	-2.16	23.70	-13.93	-9.75	0.76
32.65	-12.55	-12.74	-1.23	47.24	9.56	-22.69	-1.26	31.64	-7.15	-12.14	0.35
43.56	-2.65	-19.82	-1.20	26.44	19.51	-16.51	-0.38	37.35	5.05	-17.82	-0.75
36.70	8.31	-17.39	-2.59	24.91	31.62	-20.87	-1.25	36.37	30.70	-23.86	-1.21
35.76	30.23	-22.91	-2.01	1.79	28.24	-16.66	-0.24	23.23	32.20	-21.28	-4.56
21.95	31.75	-19.55	-5.40	-12.87	31.43	-20.16	-0.80	13.62	40.97	-23.82	-0.83
3.44	32.20	-17.63	-0.20	-25.03	18.68	-19.11	-0.62	1.67	28.41	-17.32	1.14
-12.47	30.23	-18.55	-2.47	-32.55	20.30	-22.57	-1.39	-12.92	30.68	-20.37	-6.06
-23.78	20.76	-18.29	-3.24	-34.46	4.11	-20.38	-4.42	-34.79	16.75	-22.99	-4.19

-30.72	12.27	-18.97	-3.31	-39.70	-15.17	-22.89	-1.01	-42.28	12.18	-25.43	-1.03
-30.87	3.23	-17.76	-1.29	-24.55	-27.95	-18.60	-1.62	-35.98	-7.70	-20.55	-0.12
-32.27	-15.63	-19.24	-1.35	-22.29	-37.24	-21.79	-1.49	-34.61	-23.97	-22.47	-0.66
-19.37	-27.87	-17.18	1.41	-8.85	-38.06	-18.79	-0.68	-18.92	-29.73	-17.93	-1.39
-12.42	-40.48	-20.50	-5.07	3.84	-34.58	-15.85	0.70	-11.08	-38.41	-19.32	-2.54
2.63	-41.04	-18.35	-0.82	7.88	-46.14	-21.54	-0.36	6.17	-50.23	-23.10	-2.14
7.30	-48.14	-22.63	-0.38	15.54	-49.85	-23.91	0.12	3.72	-36.29	-16.61	-1.56
15.87	-50.66	-23.90	0.16	28.00	-8.66	-12.52	-0.66	13.09	-39.97	-18.89	0.62
11.88	-24.83	-11.88	-0.04	32.75	-0.73	-17.22	-0.18	32.51	-16.77	-16.02	-1.64
28.73	0.08	-14.91	-0.29	34.57	11.28	-20.28	-2.37	38.24	-6.71	-17.61	0.21
41.08	13.00	-23.26	-0.40	32.48	27.32	-25.07	-1.85	41.58	12.14	-24.61	-1.35
27.60	13.34	-17.06	1.25	15.74	39.06	-26.18	-1.43	26.09	10.31	-16.27	-1.29
19.52	38.93	-25.89	-0.43	6.04	35.47	-23.20	-1.50	24.66	20.32	-19.64	-3.26
-0.74	41.86	-26.07	-0.46	-8.59	39.88	-27.34	-1.45	8.21	32.35	-22.60	-1.55
-9.60	38.05	-24.05	-3.22	-18.79	34.17	-26.35	-0.93	-6.36	41.55	-28.54	-1.00
-21.28	30.50	-24.68	0.06	-13.45	19.77	-18.13	-0.27	-10.07	21.43	-18.40	2.61
-31.22	19.08	-23.80	1.55	-35.95	11.12	-25.91	2.24	-21.32	24.87	-23.81	-1.88
-37.67	16.12	-26.94	-0.02	-29.08	-9.45	-20.64	0.05	-19.35	16.06	-19.41	0.58
-21.37	-35.32	-23.63	-1.41	-27.63	-21.40	-22.10	-2.49	-34.23	6.52	-23.90	1.10
-10.10	-46.19	-25.77	-0.81	-14.80	-42.15	-24.87	-2.95	-22.52	-21.84	-19.69	-3.43
-5.97	-32.15	-18.34	-2.26	-4.47	-29.88	-16.78	-0.41	-19.82	-40.30	-26.11	-1.79
3.22	-24.24	-12.31	1.29	4.31	-21.02	-10.76	-0.76	-2.98	-23.97	-13.15	0.16
7.31	-34.71	-18.61	0.03	6.73	-27.48	-14.54	-0.94	-3.21	-43.04	-23.79	-0.96
6.90	-18.08	-9.43	1.36	20.48	-12.08	-11.17	0.38	5.95	-22.68	-11.62	0.01
18.59	-11.16	-10.20	-1.48	36.23	4.04	-22.30	-0.82	11.75	-45.29	-24.36	0.62
33.04	15.27	-22.90	-0.81	28.55	6.08	-18.38	-2.39	15.05	-21.26	-12.51	-0.32
27.04	24.60	-24.72	-1.05	15.06	26.46	-22.58	-1.63	28.12	0.65	-17.43	-4.33
12.46	27.29	-21.66	-2.84	12.42	32.33	-25.93	-0.74	32.49	11.09	-22.76	-1.05
8.38	35.08	-25.18	-1.11	-4.06	31.02	-24.88	-1.51	15.70	26.27	-22.70	-4.36
-2.05	24.04	-19.13	1.36	-3.39	20.00	-17.95	0.03	-4.49	31.74	-25.96	-1.10
-12.25	18.90	-18.84	-0.04	-19.42	24.03	-25.80	-0.79	-1.76	19.76	-18.14	-1.35
-17.74	22.85	-23.23	-2.30	-31.30	16.46	-28.79	0.83	-25.85	16.56	-26.15	-1.38
-28.59	-4.15	-21.92	1.14	-25.38	0.65	-21.40	-1.16	-24.98	1.42	-21.50	0.08
-23.66	-20.46	-22.59	-0.56	-14.24	-29.36	-22.26	1.37	-15.64	-33.33	-24.84	0.10
-5.74	-23.37	-15.79	0.64	-1.85	-40.89	-25.73	-1.28	-8.84	-28.34	-19.54	-0.76
25.68	-12.57	-15.67	-0.75	-1.16	-34.51	-21.89	0.14	2.86	-28.11	-17.05	0.06
19.03	15.69	-18.76	-0.66	11.69	-21.36	-13.19	0.40	23.35	-5.67	-13.84	0.89
19.12	22.69	-23.75	1.71	23.70	22.44	-26.33	-0.22	5.33	24.24	-23.70	-0.30
4.72	25.88	-23.01	0.78	5.72	26.54	-24.19	1.48	-9.34	13.78	-19.17	0.50
-8.62	27.58	-26.28	-0.31	-23.05	17.02	-27.66	-2.84	-33.35	12.27	-33.07	1.23

-28.59	8.95	-27.19	1.86	-27.44	7.47	-27.20	2.42	-26.93	-13.90	-26.81	-0.57
-20.38	2.02	-19.92	-0.53	-4.36	-20.08	-15.34	-0.43	16.49	-9.64	-10.78	0.23
-14.11	-28.88	-25.23	-0.20	22.90	12.99	-23.32	0.35	26.31	16.73	-28.58	1.13
25.40	5.70	-20.78	-1.51	-8.43	13.22	-19.99	0.13	20.11	22.80	-29.52	0.86
12.71	20.75	-22.43	-4.17	-14.99	14.67	-25.28	1.36	-24.17	8.69	-29.67	2.64
-23.69	17.19	-30.71	-0.72	-25.33	-13.85	-29.34	-0.13	-21.77	-6.70	-24.95	-1.83
-25.99	-11.49	-27.71	-0.90	-17.21	-19.09	-24.85	-0.23	-16.59	-24.00	-27.00	1.83
-11.14	-22.23	-21.89	3.12	19.04	-1.17	-16.07	-2.00	-7.22	-20.11	-18.70	-0.05
18.51	-2.15	-15.59	0.55	13.95	16.81	-24.46	-4.99	23.81	8.29	-25.14	3.00
-2.71	12.94	-18.31	-2.46	-23.89	11.82	-34.68	0.29	-14.78	13.73	-28.76	0.18
-18.64	9.75	-27.33	2.57	-15.38	-7.02	-21.90	-7.23	-14.82	0.69	-21.97	-4.04
-14.36	-7.56	-20.09	-4.04	-9.34	-18.51	-21.82	-0.44	-15.79	-14.49	-24.59	-2.46
-16.11	-14.99	-24.77	-2.25	26.70	7.77	-29.99	0.81	18.23	1.16	-18.82	-1.95
5.40	17.93	-25.39	0.65	2.81	11.26	-19.17	-0.01	12.26	12.86	-23.90	-3.16
-9.75	11.21	-23.96	2.44	-18.21	2.61	-28.77	0.78	1.93	8.93	-17.59	-2.34
-5.92	-14.26	-17.90	-1.97	20.14	4.28	-24.52	-0.51	6.94	-13.05	-11.60	-0.80
13.29	6.45	-18.68	-2.01	19.08	14.91	-32.70	0.32	12.56	-2.06	-12.69	0.80
-17.57	1.68	-30.25	0.16	5.41	17.39	-29.85	-0.10	12.20	17.60	-32.64	0.67
2.86	-15.83	-17.14	-0.30	-7.79	-6.85	-18.49	-3.65	-15.36	3.99	-31.03	-0.46
9.46	-9.53	-11.15	-0.55	11.21	6.21	-20.26	0.41	-7.75	-6.59	-18.33	-3.11
-10.95	0.27	-23.62	-2.71	-8.58	6.18	-27.58	0.92	-6.60	6.18	-25.51	3.34
4.06	6.91	-18.97	-4.99	8.56	-3.68	-7.63	-0.38	9.58	4.56	-20.81	-0.60
-5.29	-4.45	-18.15	-5.50	2.97	-10.73	-13.50	-1.30	0.37	-13.80	-18.74	-0.85
-3.51	4.32	-28.16	1.26	-0.55	2.72	-22.98	-1.07	-3.75	-1.58	-24.10	-2.52
7.69	-1.24	-15.65	4.14	6.98	-0.98	-16.77	-0.42	6.11	-2.33	-17.68	0.43

Table S2. Distribution of macroH2A1 and canonical cores in fibers with 50% macroH2A1 and 50% canonical cores. 1 means the core is macroH2A1 and 0 means the core is canonical.

Core #	Short NRL	Medium NRL	Medium NRL+NFR
1	1	1	0
2	0	0	1
3	0	0	1
4	0	0	1
5	1	1	1
6	1	1	0
7	1	1	0
8	1	1	1
9	1	1	1
10	0	0	1

11	0	0	1
12	1	1	0
13	1	1	0
14	1	1	1
15	0	0	0
16	1	1	0
17	1	1	1
18	0	0	0
19	0	0	0
20	0	0	0
21	1	1	0
22	0	0	0
23	0	0	0
24	0	0	1
25	1	1	0
26	0	0	0
27	1	1	0
28	0	0	1
29	0	0	1
30	0	0	0
31	0	0	1
32	1	1	0
33	0	0	0
34	0	0	1
35	1	1	1
36	0	0	0
37	1	1	0
38	0	0	0
39	0	0	0
40	1	1	0
41	1	1	0
42	0	0	1
43	0	0	0
44	0	0	1
45	1	1	0
46	0	0	1
47	0	0	1
48	1	1	1
49	0	0	0
50	0	0	0

51	1	1	1
52	1	1	1
53	1	1	0
54	1	1	1
55	0	0	0
56	1	1	1
57	1	1	0
58	0	0	1
59	0	0	0
60	1	1	0
61	1	1	0
62	1	1	0
63	1	1	1
64	1	1	0
65	1	1	0
66	1	1	0
67	1	1	1
68	1	1	0
69	0	0	1
70	1	1	1
71	1	1	1
72	1	1	1
73	0	0	1
74	1	1	0
75	0	0	1
76	1	1	1
77	0	0	1
78	1	1	0
79	0	0	1
80	0	0	1
81	1	1	0
82	0	0	1
83	0	0	0
84	0	0	1
85	0	0	1
86	0	0	1
87	0	0	1
88	0	0	
89	1	1	
90	1	1	

91	0	0
92	0	0
93	0	0
94	1	1
95	1	1
96	0	0
97	0	0
98	1	1
99	1	1
100	1	1

Table S3. List of linker DNA length in the HTT gene. We show the healthy version of the gene that has 24 beads on the first linker DNA. The HD version of the gene is equal except for having 36 DNA beads on the first linker DNA to incorporate the CAG expansion.

Core	DNA Beads	bp
1	24	220.588235
2	2	26.4705882
3	2	26.4705882
4	7	70.5882353
5	4	44.1176471
6	2	26.4705882
7	2	26.4705882
8	2	26.4705882
9	2	26.4705882
10	2	26.4705882
11	36	326.470588
12	36	326.470588
13	2	26.4705882
14	12	114.705882
15	2	26.4705882
16	5	52.9411765
17	24	220.588235
18	5	52.9411765
19	2	26.4705882
20	12	114.705882
21	5	52.9411765
22	2	26.4705882
23	7	70.5882353
24	36	326.470588

25	18	167.647059
26	12	114.705882
27	3	35.2941176
28	7	70.5882353
29	2	26.4705882
30	2	26.4705882
31	4	44.1176471
32	2	26.4705882
33	4	44.1176471
34	6	61.7647059
35	6	61.7647059
36	24	220.588235
37	2	26.4705882
38	4	44.1176471
39	12	114.705882
40	7	70.5882353
41	2	26.4705882
42	2	26.4705882
43	5	52.9411765
44	2	26.4705882
45	7	70.5882353
46	7	70.5882353
47	18	167.647059
48	2	26.4705882
49	5	52.9411765
50	2	26.4705882
51	2	26.4705882
52	12	114.705882
53	12	114.705882
54	2	26.4705882
55	4	44.1176471
56	18	167.647059
57	6	61.7647059
58	12	114.705882
59	12	114.705882
60	12	114.705882
61	12	114.705882
62	24	220.588235
63	2	26.4705882
64	2	26.4705882

65	2	26.4705882
66	12	114.705882
67	2	26.4705882
68	12	114.705882
69	2	26.4705882
70	18	167.647059
71	2	26.4705882
72	7	70.5882353
73	12	114.705882
74	7	70.5882353
75	2	26.4705882
76	2	26.4705882
77	8	79.4117647
78	8	79.4117647
79	2	26.4705882
80	30	273.529412
81	12	114.705882
82	18	167.647059
83	18	167.647059
84	2	26.4705882
85	8	79.4117647
86	12	114.705882
87	8	79.4117647
88	2	26.4705882
89	18	167.647059
90	12	114.705882
91	8	79.4117647
92	2	26.4705882
93	36	326.470588
94	2	26.4705882
95	8	79.4117647
96	2	26.4705882
97	12	114.705882
98	18	167.647059
99	18	167.647059
100	2	26.4705882
101	18	167.647059
102	12	114.705882
103	2	26.4705882
104	2	26.4705882

105	2	26.4705882
106	2	26.4705882
107	2	26.4705882
108	2	26.4705882
109	12	114.705882
110	2	26.4705882
111	2	26.4705882
112	2	26.4705882
113	2	26.4705882
114	2	26.4705882
115	24	220.588235
116	5	52.9411765
117	12	114.705882
118	12	114.705882
119	12	114.705882
120	2	26.4705882
121	6	61.7647059
122	3	35.2941176
123	12	114.705882
124	3	35.2941176
125	12	114.705882
126	6	61.7647059
127	7	70.5882353
128	24	220.588235
129	2	26.4705882
130	7	70.5882353
131	12	114.705882
132	12	114.705882
133	18	167.647059
134	2	26.4705882
135	2	26.4705882
136	6	61.7647059
137	12	114.705882
138	2	26.4705882
139	2	26.4705882
140	2	26.4705882
141	5	52.9411765
142	4	44.1176471
143	6	61.7647059
144	2	26.4705882

145	4	44.1176471
146	5	52.9411765
147	6	61.7647059
148	5	52.9411765
149	2	26.4705882
150	12	114.705882
151	2	26.4705882
152	18	167.647059
153	12	114.705882
154	5	52.9411765
155	2	26.4705882
156	7	70.5882353
157	2	26.4705882
158	12	114.705882
159	3	35.2941176
160	7	70.5882353
161	6	61.7647059
162	12	114.705882
163	2	26.4705882
164	2	26.4705882
165	3	35.2941176
166	8	79.4117647
167	7	70.5882353
168	2	26.4705882
169	12	114.705882
170	12	114.705882
171	12	114.705882
172	2	26.4705882
173	2	26.4705882
174	12	114.705882
175	12	114.705882
176	12	114.705882
177	36	326.470588
178	2	26.4705882
179	12	114.705882
180	2	26.4705882
181	4	44.1176471
182	2	26.4705882
183	3	35.2941176
184	2	26.4705882

185	7	70.5882353
186	2	26.4705882
187	7	70.5882353
188	12	114.705882
189	2	26.4705882
190	4	44.1176471
191	7	70.5882353
192	18	167.647059
193	2	26.4705882
194	5	52.9411765
195	2	26.4705882
196	2	26.4705882
197	2	26.4705882
198	12	114.705882
199	4	44.1176471
200	6	61.7647059
201	2	26.4705882
202	2	26.4705882
203	12	114.705882
204	12	114.705882
205	2	26.4705882
206	12	114.705882
207	4	44.1176471
208	5	52.9411765
209	3	35.2941176
210	4	44.1176471
211	2	26.4705882
212	36	326.470588
213	7	70.5882353
214	2	26.4705882
215	3	35.2941176
216	3	35.2941176
217	2	26.4705882
218	30	273.529412
219	24	220.588235
220	2	26.4705882
221	2	26.4705882
222	2	26.4705882
223	6	61.7647059
224	8	79.4117647

225	2	26.4705882
226	2	26.4705882
227	12	114.705882
228	2	26.4705882
229	24	220.588235
230	2	26.4705882
231	24	220.588235
232	2	26.4705882
233	12	114.705882
234	24	220.588235
235	2	26.4705882
236	4	44.1176471
237	2	26.4705882
238	2	26.4705882
239	7	70.5882353
240	4	44.1176471
241	3	35.2941176
242	12	114.705882
243	2	26.4705882
244	18	167.647059
245	2	26.4705882
246	12	114.705882
247	2	26.4705882
248	12	114.705882
249	7	70.5882353
250	2	26.4705882
251	30	273.529412
252	4	44.1176471
253	2	26.4705882
254	8	79.4117647
255	2	26.4705882
256	2	26.4705882
257	5	52.9411765
258	8	79.4117647
259	12	114.705882
260	7	70.5882353
261	4	44.1176471
262	2	26.4705882
263	2	26.4705882
264	5	52.9411765

265	2	26.4705882
266	4	44.1176471
267	2	26.4705882
268	12	114.705882
269	8	79.4117647
270	12	114.705882
271	6	61.7647059
272	12	114.705882
273	12	114.705882
274	36	326.470588
275	24	220.588235
276	8	79.4117647
277	12	114.705882
278	2	26.4705882
279	12	114.705882
280	18	167.647059
281	12	114.705882
282	2	26.4705882
283	6	61.7647059
284	2	26.4705882
285	18	167.647059
286	12	114.705882
287	2	26.4705882
288	12	114.705882
289	7	70.5882353
290	12	114.705882
291	2	26.4705882
292	12	114.705882
293	18	167.647059
294	12	114.705882
295	2	26.4705882
296	2	26.4705882
297	12	114.705882
298	12	114.705882
299	18	167.647059
300	12	114.705882
301	2	26.4705882
302	12	114.705882
303	18	167.647059
304	6	61.7647059

305	7	70.5882353
306	8	79.4117647
307	12	114.705882
308	5	52.9411765
309	4	44.1176471
310	2	26.4705882
311	2	26.4705882
312	18	167.647059
313	18	167.647059
314	3	35.2941176
315	12	114.705882
316	6	61.7647059
317	2	26.4705882
318	18	167.647059
319	2	26.4705882
320	2	26.4705882
321	2	26.4705882
322	8	79.4117647
323	2	26.4705882
324	2	26.4705882
325	2	26.4705882
326	12	114.705882
327	18	167.647059
328	2	26.4705882
329	18	167.647059
330	2	26.4705882
331	2	26.4705882
332	2	26.4705882
333	8	79.4117647
334	8	79.4117647
335	2	26.4705882
336	2	26.4705882
337	3	35.2941176
338	12	114.705882
339	4	44.1176471
340	12	114.705882
341	8	79.4117647
342	18	167.647059
343	2	26.4705882
344	24	220.588235

345	18	167.647059
346	5	52.9411765
347	24	220.588235
348	2	26.4705882
349	8	79.4117647
350	2	26.4705882
351	2	26.4705882
352	24	220.588235
353	6	61.7647059
354	5	52.9411765
355	7	70.5882353
356	12	114.705882
357	2	26.4705882
358	2	26.4705882
359	7	70.5882353
360	12	114.705882
361	5	52.9411765
362	2	26.4705882
363	4	44.1176471
364	12	114.705882
365	12	114.705882
366	12	114.705882
367	36	326.470588
368	2	26.4705882
369	2	26.4705882
370	4	44.1176471
371	3	35.2941176
372	2	26.4705882
373	2	26.4705882
374	2	26.4705882
375	2	26.4705882
376	3	35.2941176
377	2	26.4705882
378	7	70.5882353
379	12	114.705882
380	12	114.705882
381	2	26.4705882
382	2	26.4705882
383	3	35.2941176
384	6	61.7647059

385	24	220.588235
386	18	167.647059
387	18	167.647059
388	18	167.647059
389	2	26.4705882
390	3	35.2941176
391	12	114.705882
392	12	114.705882
393	18	167.647059
394	2	26.4705882
395	2	26.4705882
396	2	26.4705882
397	2	26.4705882
398	2	26.4705882
399	6	61.7647059
400	2	26.4705882
401	12	114.705882
402	6	61.7647059
403	8	79.4117647
404	2	26.4705882
405	12	114.705882
406	3	35.2941176
407	6	61.7647059
408	18	167.647059
409	18	167.647059
410	2	26.4705882
411	12	114.705882
412	24	220.588235
413	12	114.705882
414	7	70.5882353
415	18	167.647059
416	4	44.1176471
417	12	114.705882
418	2	26.4705882
419	7	70.5882353
420	12	114.705882
421	12	114.705882
422	2	26.4705882
423	8	79.4117647
424	7	70.5882353

425	18	167.647059
426	2	26.4705882
427	24	220.588235
428	18	167.647059
429	5	52.9411765
430	24	220.588235
431	18	167.647059
432	2	26.4705882
433	2	26.4705882
434	24	220.588235
435	2	26.4705882
436	30	273.529412
437	36	326.470588
438	12	114.705882
439	24	220.588235
440	2	26.4705882
441	8	79.4117647
442	6	61.7647059
443	2	26.4705882
444	4	44.1176471
445	4	44.1176471
446	12	114.705882
447	2	26.4705882
448	30	273.529412
449	7	70.5882353
450	12	114.705882
451	24	220.588235
452	6	61.7647059
453	4	44.1176471
454	30	273.529412
455	2	26.4705882
456	12	114.705882
457	2	26.4705882
458	2	26.4705882
459	12	114.705882
460	4	44.1176471
461	5	52.9411765
462	3	35.2941176
463	12	114.705882
464	12	114.705882

465	12	114.705882
466	18	167.647059
467	12	114.705882
468	4	44.1176471
469	2	26.4705882
470	24	220.588235
471	2	26.4705882
472	4	44.1176471
473	5	52.9411765
474	36	326.470588
475	2	26.4705882
476	4	44.1176471
477	12	114.705882
478	12	114.705882
479	2	26.4705882
480	7	70.5882353
481	2	26.4705882
482	18	167.647059
483	2	26.4705882
484	12	114.705882
485	2	26.4705882
486	30	273.529412
487	8	79.4117647
488	2	26.4705882
489	7	70.5882353
490	12	114.705882
491	18	167.647059
492	12	114.705882
493	18	167.647059
494	2	26.4705882
495	7	70.5882353
496	12	114.705882
497	6	61.7647059
498	2	26.4705882
499	2	26.4705882
500	2	26.4705882
501	2	26.4705882
502	7	70.5882353
503	18	167.647059
504	2	26.4705882

505	12	114.705882
506	2	26.4705882
507	2	26.4705882
508	2	26.4705882
509	6	61.7647059
510	18	167.647059
511	2	26.4705882
512	4	44.1176471
513	24	220.588235
514	8	79.4117647
515	2	26.4705882
516	8	79.4117647
517	2	26.4705882
518	24	220.588235
519	12	114.705882
520	2	26.4705882
521	12	114.705882
522	30	273.529412
523	2	26.4705882
524	4	44.1176471
525	2	26.4705882
526	2	26.4705882
527	12	114.705882
528	3	35.2941176
529	12	114.705882
530	2	26.4705882
531	24	220.588235
532	2	26.4705882
533	3	35.2941176
534	2	26.4705882
535	12	114.705882
536	8	79.4117647
537	2	26.4705882
538	4	44.1176471
539	12	114.705882
540	7	70.5882353
541	2	26.4705882
542	24	220.588235
543	7	70.5882353
544	2	26.4705882

545	24	220.588235
546	2	26.4705882
547	4	44.1176471
548	5	52.9411765
549	12	114.705882
550	2	26.4705882
551	2	26.4705882
552	2	26.4705882
553	6	61.7647059
554	2	26.4705882
555	12	114.705882
556	2	26.4705882
557	12	114.705882
558	5	52.9411765
559	2	26.4705882
560	6	61.7647059
561	7	70.5882353
562	12	114.705882
563	12	114.705882
564	2	26.4705882
565	8	79.4117647
566	2	26.4705882
567	2	26.4705882
568	2	26.4705882
569	2	26.4705882
570	4	44.1176471
571	12	114.705882
572	12	114.705882
573	6	61.7647059
574	5	52.9411765
575	2	26.4705882
576	3	35.2941176
577	8	79.4117647
578	12	114.705882
579	18	167.647059
580	3	35.2941176
581	3	35.2941176
582	2	26.4705882
583	12	114.705882
584	18	167.647059

585	12	114.705882
586	2	26.4705882
587	12	114.705882
588	2	26.4705882
589	12	114.705882
590	12	114.705882
591	4	44.1176471
592	2	26.4705882
593	2	26.4705882
594	24	220.588235
595	2	26.4705882
596	2	26.4705882
597	12	114.705882
598	3	35.2941176
599	2	26.4705882
600	12	114.705882
601	12	114.705882
602	12	114.705882
603	4	44.1176471
604	3	35.2941176
605	12	114.705882
606	12	114.705882
607	2	26.4705882
608	12	114.705882
609	2	26.4705882
610	2	26.4705882
611	2	26.4705882
612	2	26.4705882
613	2	26.4705882
614	12	114.705882
615	7	70.5882353
616	6	61.7647059
617	3	35.2941176
618	2	26.4705882
619	12	114.705882
620	12	114.705882
621	4	44.1176471
622	6	61.7647059
623	6	61.7647059
624	2	26.4705882

625	6	61.7647059
626	24	220.588235
627	4	44.1176471
628	2	26.4705882
629	12	114.705882
630	8	79.4117647
631	2	26.4705882
632	7	70.5882353
633	5	52.9411765
634	12	114.705882
635	12	114.705882
636	8	79.4117647
637	5	52.9411765
638	2	26.4705882
639	7	70.5882353
640	2	26.4705882
641	2	26.4705882
642	3	35.2941176
643	2	26.4705882
644	5	52.9411765
645	18	167.647059
646	36	326.470588
647	12	114.705882
648	2	26.4705882
649	5	52.9411765
650	12	114.705882
651	2	26.4705882
652	24	220.588235
653	2	26.4705882
654	7	70.5882353
655	18	167.647059
656	2	26.4705882
657	2	26.4705882
658	12	114.705882
659	6	61.7647059
660	6	61.7647059
661	2	26.4705882
662	4	44.1176471
663	8	79.4117647
664	5	52.9411765

665	5	52.9411765
666	5	52.9411765
667	12	114.705882
668	2	26.4705882
669	3	35.2941176
670	12	114.705882
671	2	26.4705882
672	4	44.1176471
673	2	26.4705882
674	12	114.705882
675	12	114.705882
676	6	61.7647059
677	8	79.4117647
678	18	167.647059
679	24	220.588235
680	12	114.705882
681	5	52.9411765
682	2	26.4705882
683	12	114.705882
684	18	167.647059
685	24	220.588235
686	2	26.4705882
687	12	114.705882
688	2	26.4705882

References

Alcalá-Vida R.; Seguin J.; Lotz C.; Molitor A. M.; Irastorza-Azcarate I.; Awada A.; Karasu N.; Bombardier A.; Cosquer B.; Skarmeta J. L. G.; Cassel J.-C.; Boutillier A.-L.; Sexton T.; and Merienne K. Age-related and disease locus-specific mechanisms contribute to early remodelling of chromatin structure in Huntington's disease mice. *Nature Comm*, 2021, 12 (1):364.
